# Ultrasound-Assisted multimodal neuromodulation via nanosystems

**DOI:** 10.1186/s12951-026-04205-8

**Published:** 2026-04-19

**Authors:** Syed Bilal Nizami, Nicola Toschi, Allegra Conti

**Affiliations:** 1 Medical Physics Section, Department of Biomedicine and Prevention, University of Rome ‘Tor Vergata’, Rome, Italy; 2 Athinoula A. Martinos Center for Biomedical Imaging, Harvard Medical School, Boston, MA USA

**Keywords:** Nanoparticles, Therapeutic ultrasound, Neurostimulation, Drug delivery, Piezoelectricity, Blood brain barrier (BBB) opening

## Abstract

Neuromodulation techniques have emerged as transformative tools for treating several neurological and psychiatric disorders, offering alternatives to traditional pharmacological approaches often hindered by the blood–brain barrier and off-target effects. While conventional modalities like deep brain stimulation, transcranial magnetic stimulation, and optogenetics have shown promise, they each face limitations in invasiveness, spatial resolution, or clinical applicability. In recent years, low-intensity ultrasound has gained attention as a noninvasive, deep-penetrating modality capable of modulating brain circuits with millimeter-sized spatial precision. This review explores the synergistic integration of ultrasound with engineered nanosystems to achieve multimodal neuromodulation such as electrical, mechanical, optical, and chemical via acoustic energy. We describe how nanoscale materials can transduce ultrasound into localized bioelectric signals, mechanical stress, light emission, or controlled drug release. These interactions enable precise, on-demand stimulation or inhibition of neuronal activity, including in deep brain regions. Experimental studies have demonstrated neuromodulatory effects across a variety of models, with applications ranging from optogenetics and drug delivery to behavioral modulation in rodents and primates. The review concludes with a critical assessment of the translational challenges such as nanoparticle delivery, biocompatibility, long-term clearance, and safety thresholds for human use while outlining promising strategies like cell-based delivery, biodegradable materials, and closed-loop control systems. These innovations highlight the potential of ultrasound-assisted nanosystems as transformative tools for precise neuromodulation in both experimental neuroscience and clinical applications.

**Graphical abstract:**

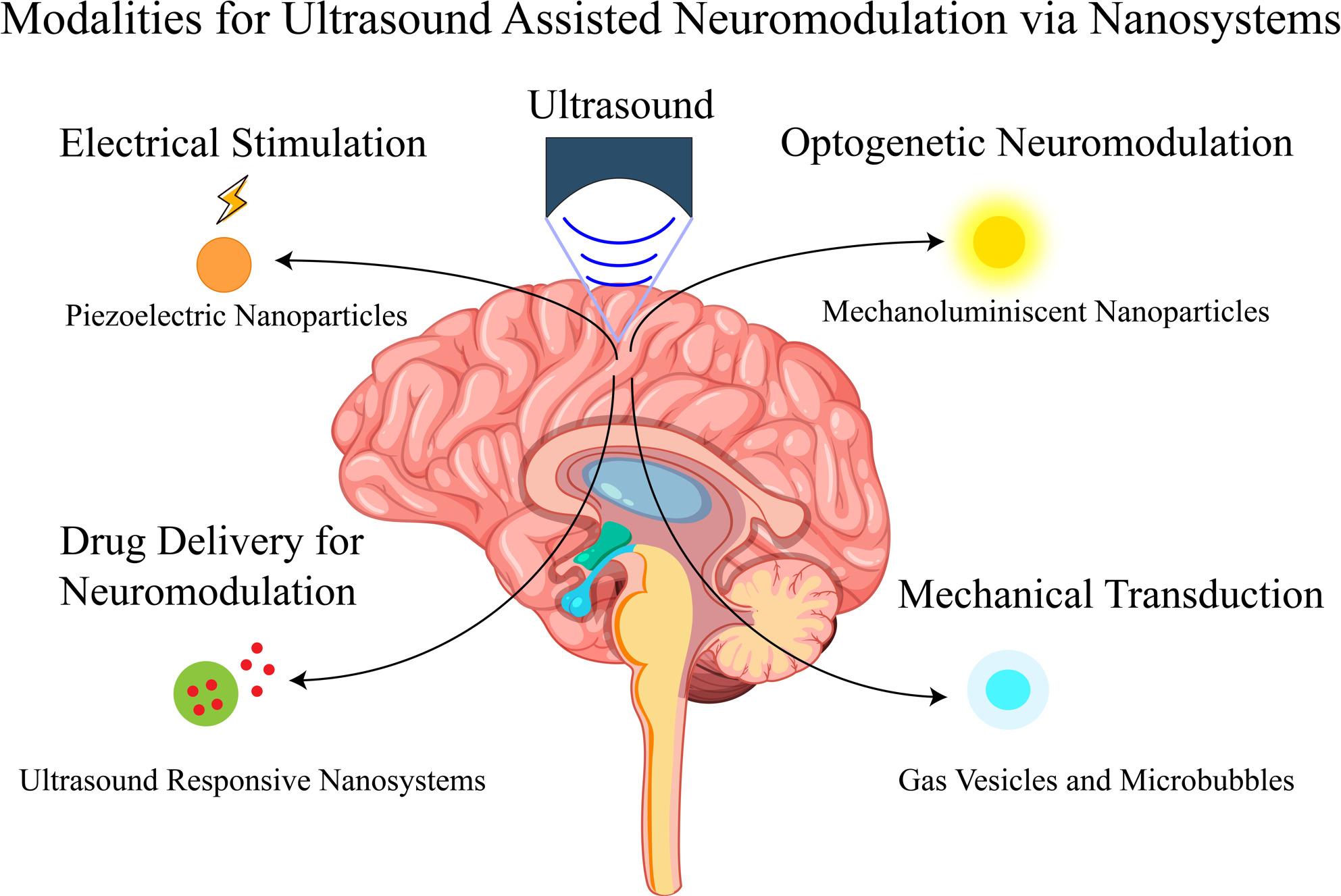

## Introduction

Neurological disorders are a leading cause of disability and the second leading cause of death worldwide​. Over the past 30 years, the number of deaths from neurological diseases increased by ~ 39%, and disability-adjusted life-years (DALYs) increased by ~ 15%​ [1]. Despite this immense burden, progress in pharmacological therapies has been sluggish, due in part to challenges like the blood–brain barrier (BBB) that limits drug delivery to the brain. These shortcomings have spurred intense interest in neuromodulation techniques as alternative or adjunct treatments for neurological and psychiatric disorders. Neuromodulation broadly refers to the targeted regulation of neural activity using external stimuli, and it can alleviate symptoms or restore function by modulating dysfunctional brain circuits.

Traditional neuromodulation modalities include electrical, magnetic, and optical stimulation. For example, deep brain stimulation (DBS) delivers electrical pulses via implanted electrodes and is FDA-approved for movement disorders like Parkinson’s disease [2]​. DBS can also alleviate severe depression​ and other conditions [3], but it requires highly invasive neurosurgery (implantation of electrodes and pulse generators) which carries risks of hemorrhage and infection [4]​. Moreover, DBS lacks cell-type specificity and can disrupt both pathological and healthy circuits, sometimes causing side effects such as speech impairment or mood changes [5]​. Noninvasive alternatives exist – transcranial direct current stimulation (tDCS) and transcranial magnetic stimulation (TMS) stimulate the brain via scalp electrodes or magnetic coils – but these have limited spatial precision and depth. tDCS induces diffuse weak electric fields (~ 0.5–2 mA) that modulate cortical excitability, yet results are variable and targeting deep structures is not possible​​ [6]. Similarly, TMS can only reliably stimulate superficial cortex and its focality worsens with depth [7]​. Optogenetics offers cell-specific control by genetically introducing light-gated ion channels (opsins) into neurons, but *in vivo* use typically needs invasive fiber-optic implants and gene therapy, hindering clinical translation​ [8].

Low-intensity (< 3 W/cm^2^) therapeutic ultrasound (US) has emerged as a powerful neuromodulation modality that can overcome many limitations of the above techniques in terms of invasiveness, spatial resolution and depth of stimulation [9]. US can be noninvasively transmitted through the skull and precisely focused to millimeter-sized regions deep in the brain. Unlike electromagnetic methods, US can reach deep nuclei, more than 10–15 cm depth of stimulation, with high spatial resolution and without surgical implants [10]. Table 1 provides a detailed comparison between US and the other neuromodulatory modalities. Initial clinical studies suggest that US neuromodulation is feasible and safe for various conditions. For example, low-intensity transcranial US has been explored for treating depression and shown promising early results​, and pilot trials in Parkinson’s disease and Alzheimer’s disease have indicated beneficial effects​ [11–13]. Furthermore, focused ultrasound (FUS) in combination with intravenously administered microbubbles (MBs) has emerged as a powerful, noninvasive approach for transiently and locally overcoming the transport restrictions imposed by the blood–brain barrier (BBB) [14]. This concept was first demonstrated by Hynynen *et al.* in 2001, who showed that applying FUS in the presence of MBs could temporarily increase vascular permeability in rabbits [15]. Mechanistically, ultrasound is a sinusoidal pressure wave, and FUS is the convergence of many ultrasound waves to a small volume, producing an amplified effect. At low acoustic intensities, gas-filled MBs within cerebral capillaries undergo regular cycles of expansion and contraction called cavitations which exert mechanical forces on adjacent endothelial walls [16]. These mechanical forces transiently disrupt the BBB by loosening endothelial tight junctions and facilitating the movement of drug molecules and nanoparticles into the brain.

Another key advantage of US is its ability to interact with a range of nanoscale agents to achieve multimodal neuromodulation. Ultrasonic energy can be converted by engineered nanosystems into secondary stimuli – electrical, mechanical, or optical cues, or into localized drug release – that then modulate neural activity. In essence, nanoparticles can act as transducers that translate the acoustic signal into a biological effect on neurons. By tailoring the nanoparticle properties, one can achieve on-demand stimulation or inhibition of specific neural circuits with higher spatial precision deep in the brain [17]. For example, US-activated nanoparticles can generate localized electric fields to depolarize neurons (electro-neuromodulation), exert mechanical forces that trigger mechanosensitive ion channels, emit light to stimulate opsin-expressing cells (sono-optogenetics), or release neuroactive drugs in specific regions.

Combining US with such functionalized nanosystems thus offers a multimodal toolkit for neural control: the ultrasound provides targetable, remote energy delivery, and the nanoparticles provide the mechanism to interface with neurons at the molecular level. Importantly, these effects can occur at the level of individual neurons or neuronal subpopulations, achieving spatiotemporal precision beyond what traditional electrodes or drugs alone can offer​​.

In this review, we first overview the fundamentals of ultrasound and the general modalities by which neural activity can be modulated (electrical, mechanical, and optical pathways). We then discuss the working principles of various US-responsive nanosystems – including piezoelectric nanomaterials, gas vesicles, and mechanoluminescent particles – detailing how they convert ultrasound into bioelectrical, biomechanical, or biochemical stimuli. Next, we survey recent experimental studies that leverage these nanosystems for neuromodulation *in vitro* and *in vivo*, highlighting breakthroughs up to 2025. Finally, we examine the challenges for clinical translation (such as delivery across the BBB, biosafety and clearance of nanoparticles, and regulatory considerations) and outline future directions for this interdisciplinary field spanning neuroscience, bioengineering, and nanomedicine. Key synthesis elements are provided in Table 1 (modality comparison), Table 2 (recommended reporting parameters), Table 3 (nanosystem class comparison), Tables 4, 5, 6 and 7 (study summaries), and Fig. 5 (translational challenges overview).

**Fig. 1:**
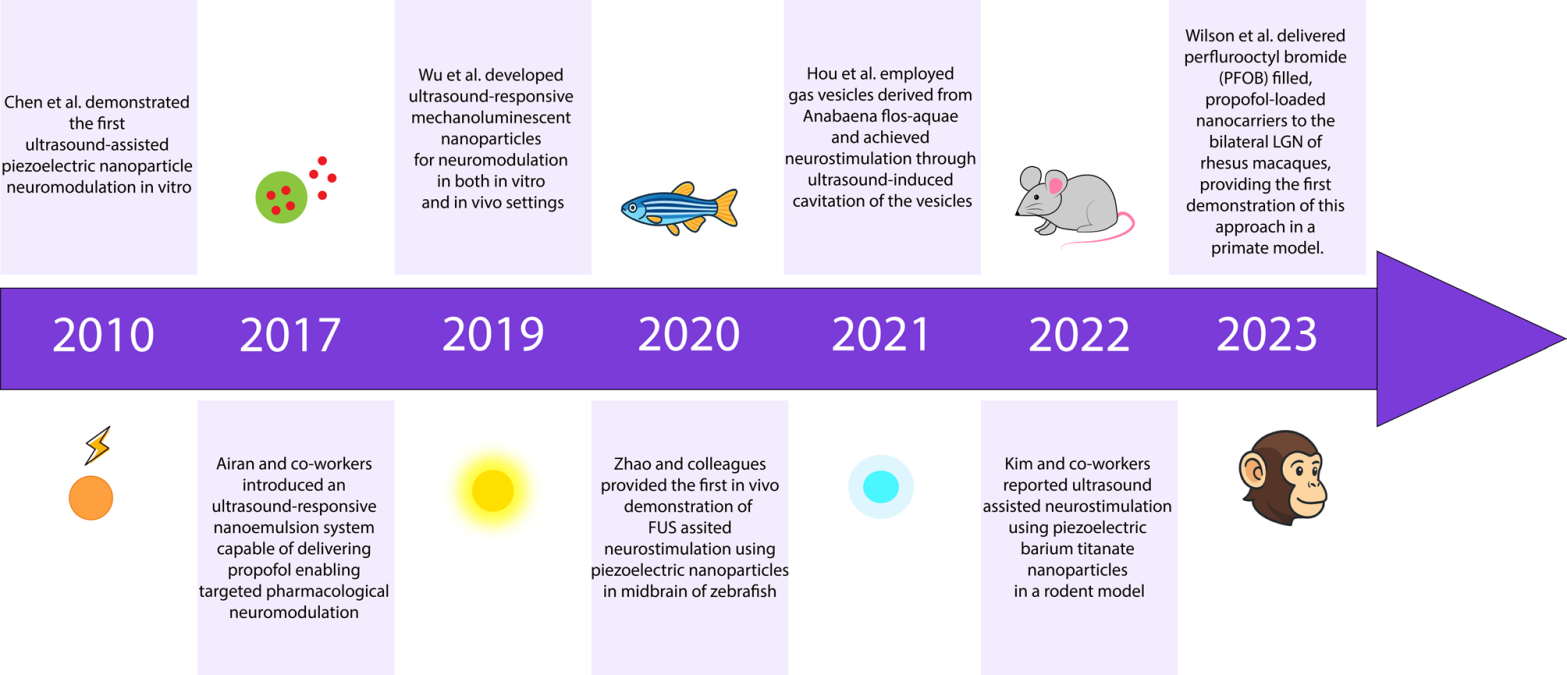
Schematic summary of key advances in focused ultrasound (FUS)–assisted neuromodulation using nanosystems, highlighting the development of various nanosystems and their progress over time

As summarized in Fig. 1, the current literature landscape on ultrasound assisted neural stimulation using nanoparticles spans multiple application areas, experimental models, and nanoparticle types. It provides a comprehensive scheme summarizing the current literature landscape on ultrasound-assisted neural stimulation using nanoparticles, highlighting the progress and distribution of research cross application areas, experimental models, and nanoparticle types within this field.

**Table 1:** Comparison of FUS with other neuromodulatory modalities (based on Bystritsky *et al.,* 2011) [167]

Parameter	Deep brain stimulation (DBS)	Transcranial current stimulation (tCS)	Transcranial magnetic stimulation (TMS)	Focused ultrasound (FUS)
Invasiveness	Invasive	Noninvasive	Noninvasive	Noninvasive
Spatial Resolution	~ 1 mm	> 500 mm Low / diffuse (cm-scale; montage-dependent)	~ 3–5 cm	1–5 mm (Depending on the frequency)
Temporal Resolution	~ 1 kHz	~ 1 kHz Low–moderate (DC/low-frequency waveforms; protocol-dependent)	µs pulses; protocol repetition typically 1–50 Hz	~ 1 kHz
Depth of stimulation	Unlimited	Primarily superficial cortex (diffuse fields)	~ 1–1.5 cm unless H-coil is used	> 10–15 cm
Closed-loop compatible	Yes	Yes	No	Yes
Reversibility	Mostly	Yes	Yes	Yes
Duration of neuromodulation (Post Treatment)	~ 5 s	24 h	~ 5 s	~ 10–40 min

## Fundamentals of ultrasound

Ultrasound refers to acoustic waves above the audible frequency range (> 20 kHz). For biomedical applications, frequencies in the range of ~ 100 kHz to several MHz are used. In brain stimulation, ultrasound typically operates at < 1–2 MHz to achieve deeper penetration with minimal attenuation. Higher frequencies correspond to shorter wavelengths, thereby enabling greater spatial resolution, as resolution is inversely proportional to wavelength. However, increasing the frequency also leads to greater acoustic attenuation when propagating through the skull. In human and large animal applications, where skull thickness poses a limiting factor, frequencies in the range of approximately 250–650 kHz are commonly employed [18]. An ultrasound transducer (often a piezoelectric element) converts electrical signals into mechanical vibrations that generate pressure waves in tissue. By using a curved transducer or an acoustic lens/array, these waves can be converged to a focal point within the brain. The result is a small sonication zone (on the order of a few millimeters or less in diameter) where acoustic energy is concentrated, while regions outside the focus receive much lower intensities [19].

**Fig. 2:**
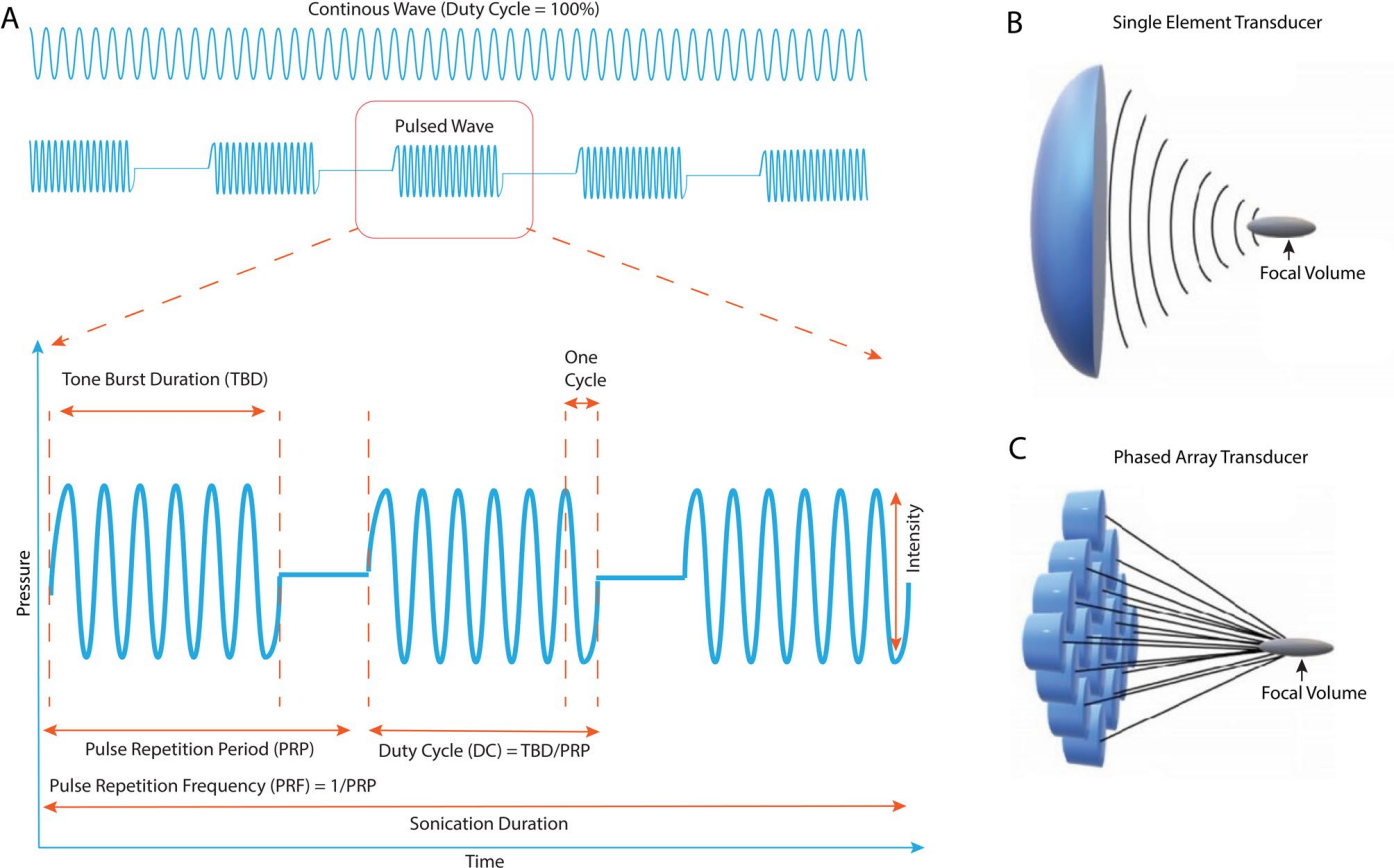
Schematic representation of **A**) a continuous wave sonication (top), a pulsed wave sonication (bottom) and FUS parameters. **C**) A single element FUS transducer consisting of a curved piezoelectric element that generates convergent acoustic waves. **D**) A phased array FUS transducer. Images **C**) and **D**) reproduced with permission via license CC BY-NC 4.0 [Yildirim *et al.*] “[Colloids, nanoparticles, and materials for imaging, delivery, ablation, and theranostics by focused ultrasound (FUS)]”, [Theranostics], © [2019], Ivyspring International Publisher

Several key parameters define an US neuromodulation protocol (Fig. 2)​​:

- Frequency: Higher frequencies produce smaller focal spots (improved spatial resolution) but are more strongly attenuated by tissue. Frequencies ~ 0.2–0.7 MHz are common for transcranial neuromodulation to balance focal precision and penetration [20].
- Intensity: Usually reported as spatial peak temporal average (I_SPTA_) and spatial peak pulse average (I_SPPA_). I_SPTA_ quantifies the average intensity over the entire duration of the sonication and I_SPPA_ represents the average intensity within a single pulse [21]. To ensure thermal safety, the US Food and Drug Administration (FDA) has established guidelines for diagnostic ultrasound imaging devices. These guidelines recommend a maximum derated I_SPTA_ of 720 mW/cm² and a maximum derated I_SPPA_ of 190 W/cm² to mitigate risks of tissue heating and thermal damage [22]. An additional guideline, IEC standard 60601-2-5 for physiotherapy ultrasound equipment, specifies an upper limit for the “effective intensity,” defined as the ratio of acoustic output power to the effective radiating area, of 3 W/cm². This “effective intensity” limit of 3 W/cm² is commonly interpreted as the maximum allowable limit for I_SPTA_. Low-intensity ultrasound used for neuromodulation is typically safe and does not cause tissue heating or damage when kept within these recommended I_SPTA_ and I_SPPA_ limits [21].
- Pulse Repetition Frequency (PRF) and Duty Cycle (DC): To stimulate the brain, US is often applied in pulses rather than continuously. Pulse Repetition Frequency (PRF- see Fig. 2) is the rate of pulses usually expressed in Hertz (Hz) and denotes the number of acoustic pulses delivered per second [23]. It is the inverse of the Pulse Repetition Period (PRP), i.e. the time interval between the start of one ultrasound pulse and the start of the next pulse. Duty cycle represents the fraction of time the ultrasound is active during each pulse. It is the ratio between the tone burst duration (TBD) to PRP, usually expressed as a percentage. A 100% duty cycle corresponds to a continuous wave without any delay between bursts [24]. DC and PRF influence the neuromodulatory outcomes, with higher duty cycles (30–100%) and higher PRF (0.1–2.8 kHz) typically producing excitatory (E) effects [25, 26] and lower duty cycles (3–5%) and lower PRF (0.03–0.1 kHz) producing inhibitory (I) effects [27, 28]. A theoretical model predicting the neuromodulation outcomes (E/I) as a function of different US parameters has been developed by Plaksin and colleagues [29]. In human studies, E/I neuromodulation is achieved by using duty cycles ranging from 3% to 50% and PRF ranging from 1 Hz to 3 kHz [30–32]. DC and PRF higher than these values can activate thermosensitive ion channels such as transient receptor potential vanilloid 1 (TRPV1) channels and consequently stimulate neural activity [33].
- Sonication Duration (SD): The overall length of an ultrasound application, from seconds up to minutes, which may be broken into bursts/trains of pulses​.
- Targeting and Beam Correction: For transcranial US, the skull can distort and attenuate ultrasound. Computational modelling or MRI-based beam correction is often employed to adjust the phase/focus and ensure sufficient pressure at the brain target​ [34].

Within the focal zone, ultrasound exerts mechanical forces on cells and tissues. Two primary bioeffects are relevant for neuromodulation: acoustic radiation forces (steady forces due to absorption of the wave, which can deform cells or trigger mechanoreceptors) and cavitation (oscillation or collapse of microbubbles in the field, which can produce micro-streaming and strong local mechanical stresses). At the low intensities used for neuromodulation, thermal effects are minimal, and mechanical interaction with cells (often called “mechanotransduction”) is believed to dominate​ [35]. Martin *et al.* (2024) provides a comprehensive consensus guideline for standardized reporting of human transcranial ultrasound stimulation (TUS), developed by the International Transcranial Ultrasound Stimulation Standardisation Taskforce (ITRUSST). The guideline addresses the variability in reporting key technical, acoustic, and dosimetric parameters in TUS studies, which has impeded reproducibility, inter-study comparisons, and meta-analyses, particularly for low-intensity neuromodulation protocols. The guideline explicitly focuses on ultrasound delivery, including transducer characteristics, drive system configuration, free-field and *in situ* acoustic parameters, pulse timing, and intensity metrics. The guideline is organized into six core reporting domains i.e., (1) transducer and drive system characteristics (2) drive system settings, (3) free-field acoustic parameters, (4) pulse timing parameters, (5) *in situ* exposure estimates (6) intensity parameters [36]. Table 2 lists key reporting parameters for *in situ* transcranial ultrasound stimulation. Neurons can respond to ultrasound through several hypothesized mechanisms – e.g. activation of mechanosensitive ion channels in the membrane, perturbation of lipid bilayer tension, or modulation of synaptic vesicle release – even without any exogenous agents​ [37]. However, the neuromodulatory effect of ultrasound alone can be modest and variable, often requiring relatively high pressures to achieve consistent excitation or inhibition *in vivo*. This is where nanosystems come into play: they can amplify or transduce the acoustic energy into a more potent stimulus for neurons, improving both the efficacy and selectivity of ultrasound neuromodulation​ [37].

**Table 2:** Key reporting parameters for *in situ* transcranial ultrasound stimulation (based on Martin *et al.*, 2024) [36]

Parameter	Definition/formula	Units	Reporting requirements (per ITRUSST)	Example values
*In-situ* through-skull pressure estimate (at target)	Derived via simulation, derating, or experimental measurement (e.g., MR-ARFI, MR thermometry).	MPa	Report the model, attenuation coefficients, and skull transmission values used. Provide both spatial-peak and target-site pressures if they differ.	Example: Free field = 0.6 MPa → skull transmission = 58% In-brain pressure ≈ 0.35 MPa.
Mechanical Index (MI)	*MI = p*_*r,0.3*_*/√f*_*₀*_ where$\:{p}_{r,0.3}$= derated peak rarefactional pressure (MPa) using attenuation 0.3 dB·cm⁻¹·MHz⁻¹ and *f*_*0 =*_ center frequency (MHz)	Unitless	Report both free-field and *in-situ* MI values if skull transmission is modeled or measured. Include derating factors and method (e.g., simulation, skull insertion loss).	Example:$\:{p}_{r}=700kPa$, derated to 230 kPa at 270 kHz MI = 0.44.
Transcranial Mechanical Index (MI_tc_)	*MI*_*tc*_*= p*_*r,α*_*/√f*_*₀*_ where$\:{p}_{r,\alpha\:}$is the *in-situ* derated pressure after skull correction.	Unitless	Used when accounting for skull attenuation. Report estimation method for$\:{p}_{r,\alpha\:}$(e.g., simulation or empirical derating) and skull parameters (attenuation, resonance).	Reported alongside MI when *in-situ* modeling is performed.
Thermal Metrics (Temperature Rise, Thermal Index, and Thermal Dose)	**Temperature Rise (ΔT)**: Experimental measurement or thermal simulation **Thermal Index (TI)**: TIC = *W/40D*, *W* = time-averaged acoustic power of the transducer in free field in mW *D* is the equivalent aperture diameter of the transducer in cm. **Thermal Dose (Cumulative Exposure Minutes - CEM)**: *CEM =*$\:{\int\:}_{t=0}^{t=final}{R}^{\left(43-T\right)}dt$ where *R* = 0.5 for *T* ≥ 43^o^ and *R* = 0.25 for *T* < 43^o^	ΔT = °C; TI = Unitless; CEM = Minutes (equivalent at 43 °C)	Report modeled and/or measured peak temperature rise, thermal index, and thermal dose where available. Include modeling parameters (tissue type, skull attenuation, duration, absorption).	NA
I_SPPA_ (Spatial-Peak Pulse-Average Intensity)	*I*_*SPPA=*_*p*^*2*^_*sp*_*/2Z* where _sp_ is the spatial-peak pressure amplitude (the amplitude of the sinusoidal pressure signal at the location of the spatial-peak) *Z* = acoustic impedance	W/cm²	Derived from estimated *in-situ* pressure. Report the acoustic impedance (Z) used for conversion.	NA
I_SPTA_ (Spatial-Peak Time-Average Intensity)	*I*_*spta*_, _*pulse train*_ = *DC*_*pulse train*_ *I*_*sppa*_	W/cm²	Report the averaging period (e.g., per pulse train or entire protocol) and specify the duty cycle used for calculation	NA
*In-situ* Through-Skull Pressure Estimate (at target)	Derived via simulation, derating, or experimental measurement (e.g., MR-ARFI or MR thermometry)	MPa	Report the model, attenuation coefficients, and skull transmission values used. Provide both spatial-peak and target-site pressures if they differ.	Free-field pressure = 0.6 MPa Skull transmission = 58% In-brain pressure ≈ 0.35 MPa.

## Mechanism of neuromodulation modalities

Neurons are excitable cells that can be influenced by various physical stimuli. These stimuli—electrical, magnetic, mechanical, optical, or chemical—can alter the membrane potential or activate specific signaling pathways, ultimately affecting neuronal activity. Each modality interacts with neurons through distinct biophysical mechanisms and offers unique advantages and limitations in terms of precision, invasiveness, and depth of penetration. In this section, we explore the underlying principles of each neuromodulation modality and how they interface with neural tissue to achieve therapeutic or experimental outcomes.

### Electrical neuromodulation: induction by electrical vs. magnetic stimuli

#### Electrical stimulation

Electrical Stimulation directly alters the membrane potential. At rest, neurons maintain a transmembrane voltage of about − 70 mV (inside relative to outside) via ionic gradients​. Depolarizing a patch of membrane to threshold (by injecting current or applying an electric field) opens voltage-gated sodium channels, triggering an action potential that propagates along the axon. Clinical electrical neuromodulation (e.g. DBS) uses electrodes to inject current into the brain tissue, depolarizing nearby neurons​. While effective, electrical stimulation lacks specificity (all neuron types near the electrode are stimulated) and the electric field can be distorted by the conductive surroundings [38]​​. Noninvasive forms like tDCS apply weak currents across the scalp; these polarize large cortical areas but with poor focality and reproducibility​.

#### Magnetic stimulation

Magnetic stimulation of neural activity is an emerging technique with potential for clinical neuromodulation, offering a non-invasive alternative to traditional methods that require implanted electrodes. Due to the low magnetic susceptibility and electrical conductivity of biological tissue, magnetic stimulation can reach deep brain areas without significant attenuation, allowing magnetic fields in the millitesla range to pass through the skull and brain with minimal interference [39]. A time-varying magnetic field (as in TMS) induces electric currents in the brain by electromagnetic induction, which in turn modulates neural activity. TMS can stimulate cortical neurons without an implanted electrode, but the rapid fall-off of induced currents limits it mostly to superficial brain regions and it cannot easily target deep structures with precision [7]​.

### Mechanical neuromodulation

Mechanical forces or vibrations can modulate neurons, especially via mechanosensitive proteins. For instance, pushing on a neuronal membrane can open stretch-sensitive ion channels (like Piezo or TRP family channels), leading to depolarization. Ultrasound provides a means to deliver such mechanical stimuli deep in tissue. A detailed review on the mechanisms between focused ultrasound and lipid membrane at the molecular level has been published by Man *et al.* [40]. Even without any added agents, low-intensity ultrasound has been shown to evoke neuronal firing or suppress activity, presumably by mechanical perturbation of membranes or mechanoreceptors​ [41]. However, neurons do not have a dedicated “ultrasound sensor,” so the effects are indirect and may require higher intensities. The introduction of acoustic-responsive nanomaterials (discussed below) greatly enhances mechanical neuromodulation by increasing the localized forces or directly coupling to mechanosensitive pathways.

Neurons possess a low elastic modulus, indicating their reduced rigidity, and contain intracellular fluids, classifying them as viscoelastic materials [42]. This viscoelastic nature enables neurons to propagate mechanical energy through viscous heat dissipation, where kinetic energy is converted into internal energy, and to store mechanical energy elastically during deformation. These elastic properties facilitate mechanical interactions at both cellular and subcellular levels, encompassing intracellular and extracellular structures, the cytoskeleton, the extracellular matrix, and cell adhesion transmembrane proteins [43]. Two primary mechanisms have been associated with the coupling between electrophysiological and mechanical processes in the neuronal membrane: changes in membrane conformational states and the activity of mechanosensitive ion channels [24].

#### Membrane conformational states

Membrane displacements may arise from voltage-induced pressure differences between intracellular and extracellular fluids, leading to alterations in membrane curvature [44]. This phenomenon is thought to involve both chemical components, such as the natural surface tension of the membrane, and electrical components, such as the energy stored in the membrane due to its capacitance, collectively described as electrowetting. Electrowetting typically refers to changes in surface tension of a liquid, such as a spherical droplet flattening under an applied electrical potential [45]. In neurons, changes in membrane potential modulate the surface tension at the interface between intracellular and extracellular fluids. To maintain constant pressure across the membrane, these shifts in surface tension necessitate changes in membrane curvature. Conformational changes in the neuronal membrane can be externally triggered by the mechanical energy of ultrasound, which induces reconfigurations in phospholipids. These reconfigurations alter the membrane’s fluidity and permeability, creating a high-energy state that forces embedded proteins and membrane lipids to adapt. This adaptation modifies the membrane’s conformational states and capacitance, ultimately influencing neural activity [46].

#### Mechanosensitive ion channels

Mechanotransduction refers to the process by which mechanical energy is converted into neural signals through specialized sensory cells. This process is facilitated by mechanosensitive ion channels. In mammals, multiple types of mechanosensitive (MS) ion channels have been identified. The transient receptor potential (TRP) channels and the K2P family, comprising six subfamilies, including TWIK (Tandem of Pore Domains in a Weak Inward-Rectifying K+ Channel) and TREK (TWIK-Related K+ Channel), play crucial roles in mechanotransduction. Key members of the K2P family, such as TREK-1, TREK-2, and TRAAK (TWIK-Related Arachidonic Acid-Activated K+ Channel), are predominantly expressed in sensory neurons and exhibit sensitivity to diverse mechanical stimuli [47]. The Piezo family constitutes another significant group of MS ion channels, playing critical roles in mechanosensory processes, development, and regulatory functions [48]. Furthermore, high-frequency ultrasound has been shown to activate MEC-4 and MEC-6 mechanosensitive ion channels, eliciting neural activity and reversal behaviors in *C. elegans* [49].

With the discovery of these MS ion channels and the knowledge of the structural and biophysical mechanisms, our understanding of mechanotransduction is advancing rapidly. The mechanism of action underlying mechanically activated ion channels is primarily attributed to the application of mechanical stimuli to the cell membrane, which generates stress distributed across various cellular components. This stress collectively modulates MS ion channels, facilitating their transition from closed to open states. Two principal models have been proposed to explain this process: the membrane force model and the tether model.

##### Membrane force model

The membrane force model describes how tension in the cell membrane directly results in area expansion of mechanosensitive proteins, without needing help from other parts like the cytoskeleton or accessory proteins. An ion channel is considered ‘inherently mechanosensitive’ if it can be mechanically activated after purification and reconstitution in a lipid bilayer. When the cell membrane is at rest, all membrane-embedded proteins experience a combination of hydrophobic and steric forces exerted by the bilayer lipids. This is called the transbilayer pressure profile [50]. An increased tension in the plasma membrane causes it to thin, resulting in a change in the local transbilayer pressure profile. This exposes hydrophobic residues of the channels that were previously embedded within the lipid bilayer. This mechanical alteration induces a tilt in the channel subunits in response to hydrophobic mismatch between their membrane-facing domains and the bilayer, producing gating movements to open the pore [51, 52].

Kefauver *et al.* described this concept in an ‘entropy driven’ model where an increase in the planar area of the bilayer lipids shifts the equilibrium such that hydrophobic forces that tend to cluster lipids overcome the forces that mediate protein–lipid interactions. Lipids that stabilize the closed state of the protein dissociate, resulting in a conformational change to compensate for the unoccupied hydrophobic pockets. An example is the structures of the two-pore potassium channels, TRAAK and TREK-1. In closed conformation, membrane lipids are bound to a fenestration below the channel pore stabilizing the channel. In an open conformation, these lipids are absent [47].

##### Tether model

The tether model, suggests that mechanosensitive proteins are tethered to the extracellular matrix (ECM), the cytoskeleton, or both. This connection allows forces acting on these cellular components to be transferred to the ion channels through a linking structure. These proteins are thought to directly interact with mechanosensitive ion channel subunits, enabling the channels to respond to mechanical stimuli effectively [53]. In this model, gating depends on the channel’s movement relative to the membrane. Adjusting a single tether shifts the channel’s position within the membrane, altering the forces that control its gating.

The difference between the membrane force model and the single-tether model lies in how the channel is activated. In the membrane force model, changes in membrane forces, such as those caused by cell swelling, drive channel opening. In contrast, the single-tether model relies on repositioning the channel, which alters the forces acting on it within the membrane. Moving the channel into or out of the membrane plane can trigger its opening [54].

### Neuromodulation via optogenetics

Neurons can be controlled by light if they are made light-sensitive, as in optogenetics. Optical neuromodulation is typically very precise (both in targeting genetically defined cell types and in timing with millisecond resolution), but visible light does not penetrate the brain well with a penetration depth of less than 1 mm and a spatial resolution below 100 μm [55]. Therefore, fiber-optic implants or transcranial LED devices are needed, and clinical use is limited by the need for genetic modification to express light-gated channels (e.g. Channelrhodopsin) [56]​. An intriguing solution is to use ultrasound to generate light inside the brain via mechanoluminescent nanoparticles (which this review primarily focuses on), enabling sono-optogenetics without external fiber optics​​ [57]. A range of opsins are now available that respond to wavelengths spanning from infrared to ultraviolet. The penetration of light through brain tissue varies as a function of wavelength [58]. Red light penetrates tissue to the greatest extent, up to 3 mm below the surface of the brain [59], primarily due to reduced haemoglobin absorption [60]. This property is advantageous when targeting a larger population of cells or reaching neurons located deeper within the brain. However, the use of infrared light is limited by the absorption spectra of opsins, as the majority of currently available opsins respond predominantly to light within the blue/green spectrum [61]. To address the problem of invasiveness, Chen *et al*. developed a noninvasive optogenetic technique that uses radioluminescent nanophosphors (XEOL NPs) as *in situ* light sources to convert externally applied X-rays into visible red light, eliminating the need for implanted optical fibers. These Gd₂(WO₄)₃:Eu nanoparticles emit light around 610 nm, which precisely activates red-shifted opsins (ReaChR) expressed in targeted cortical neurons [62].

### Chemical neuromodulation

Lastly, neurons are modulated by neurochemicals (neurotransmitters, drugs). Focused ultrasound (FUS) can be used to deliver chemicals in a targeted manner – for example, by opening the BBB or by releasing a drug from a carrier at a specific location​​. This modality is particularly attractive for leveraging the vast array of existing neuropharmacological agents but in a spatially controlled fashion, minimizing off-target effects [63].

Ultrasound can interface with all of the above modalities via appropriate nanosystems. The graphical abstract illustrates this multimodal approach: a FUS transducer targets a brain region where systemically delivered nanoparticles have accumulated. Depending on the nanoparticle design, the FUS exposure can electrically stimulate neurons (through piezoelectric charge generation), mechanically stimulate neurons (through acoustic forces amplified by microbubbles or gas vesicles), optically stimulate neurons (through FUS-induced light emission from mechanoluminescent particles), or chemically modulate neurons (through localized drug release). In the following sections, we examine each class of FUS-responsive nanosystem in detail, explaining how they work and reviewing the latest experimental demonstrations of neuromodulation achieved with them.

## Ultrasound-responsive nanosystems: working principles, design, and applications

Various nanostructured materials have been engineered to respond to ultrasound and in turn produce stimuli that affect neuronal excitability (Table 3). The primary mechanisms by which these acoustic nanotransducers operate are: (1) converting acoustic pressure into electrical charges (piezoelectric effect), (2) converting acoustic energy into mechanical motion or forces (e.g. via bubble oscillation or deformation of structures), or (3) converting acoustic energy into light (mechanoluminescence). A fourth mechanism is (4) ultrasound-triggered chemical release (in drug-loaded nanoparticles). Below, we describe the transduction mechanisms and the types of nanosystems that exhibit them. We also reviewed experimental studies that have applied these systems for neuromodulation. These studies span from *in vitro* neuron cultures to living animals, and they highlight the progress in achieving controlled stimulation or inhibition of neural activity using US+nano approaches. We organize the discussion by the type of nanosystem and modality.

**Table 3:** Comparison of nanosystem classes used with low-intensity ultrasound for neuromodulation

Nanosystem class	Mechanism	Key advantages	Main limitations	Highest evidence level	Readiness for real applications
Piezoelectric Nanoparticles	Convert ultrasound-induced mechanical strain into local electric fields (piezoelectric effect) that depolarize neurons or stimulate excitable tissue.	• Precise electrical neuromodulation without electrodes • Deep, noninvasive stimulation • Tunable with ultrasound parameters • Can promote neuroregeneration	• Potential cytotoxicity (esp. lead-based PZT) • Localization/delivery challenges • Long-term clearance and aggregation concerns	Rodents	Mid term
Gas Vesicles / Nanobubbles / Microbubbles	Oscillation of gas vesicles or bubbles under ultrasound produces mechanical stress on neuronal membranes, activating mechanosensitive ion channels (e.g. Piezo, TRP).	• Sensitive to low-intensity US • High spatial precision (< 1 mm) • Can be genetically targeted (acoustic reporter genes) • Biodegradable and reversible stimulation	• Potential immune response (protein shells) • Pressure-dependent collapse can cause haemorrhages	Rodents	Mid term
Mechanoluminescent Nanoparticles	Convert ultrasound-induced mechanical stress into light (mechanoluminescence) that activates light-sensitive opsins in neurons (sono-optogenetics).	• Enables deep-brain optogenetic control • Fully wireless and implant-free • Combines optogenetic specificity with FUS depth • High spatiotemporal control	• Requires pre-charging or high US intensity • Low photon yield • Potential heavy-metal toxicity • Complex material synthesis	Rodents	Long term
Ultrasound-Triggered Drug Delivery Nanosystems	Ultrasound triggers mechanical disruption, cavitation, or phase-change to release neuromodulatory drugs or open BBB for targeted chemical neuromodulation.	• Enables localized delivery of neurostimulatory/inhibitory agents • Applicable to diverse drugs	• Possible off-target effects if cavitation uncontrolled	Non-human primate	Near term

### Piezoelectric nanosystems for electric field generation

#### Working principle

Piezoelectric materials generate an electric polarization when mechanically stressed and conversely deform when an electric field is applied ​​ [64]. In essence, they couple mechanical and electrical energy. When an ultrasound wave impinges on a piezoelectric nanoparticle, the pressure oscillations produce periodic deformations in the particle. This induces an oscillating electric field (voltage) across the material by the direct piezoelectric effect​ [65]. If the nanoparticle is in contact with or very near a neuron, these electric fields can influence the neuron’s membrane potential. A single nanoparticle can act like a nano-capacitor that emits alternating charges on its surface in response to ultrasound vibrations​. Clusters or films of piezoelectric nanoparticles can thus create localized electric stimuli inside tissue, effectively functioning as wireless micro-electrodes activated by sound.

**Fig. 3:**
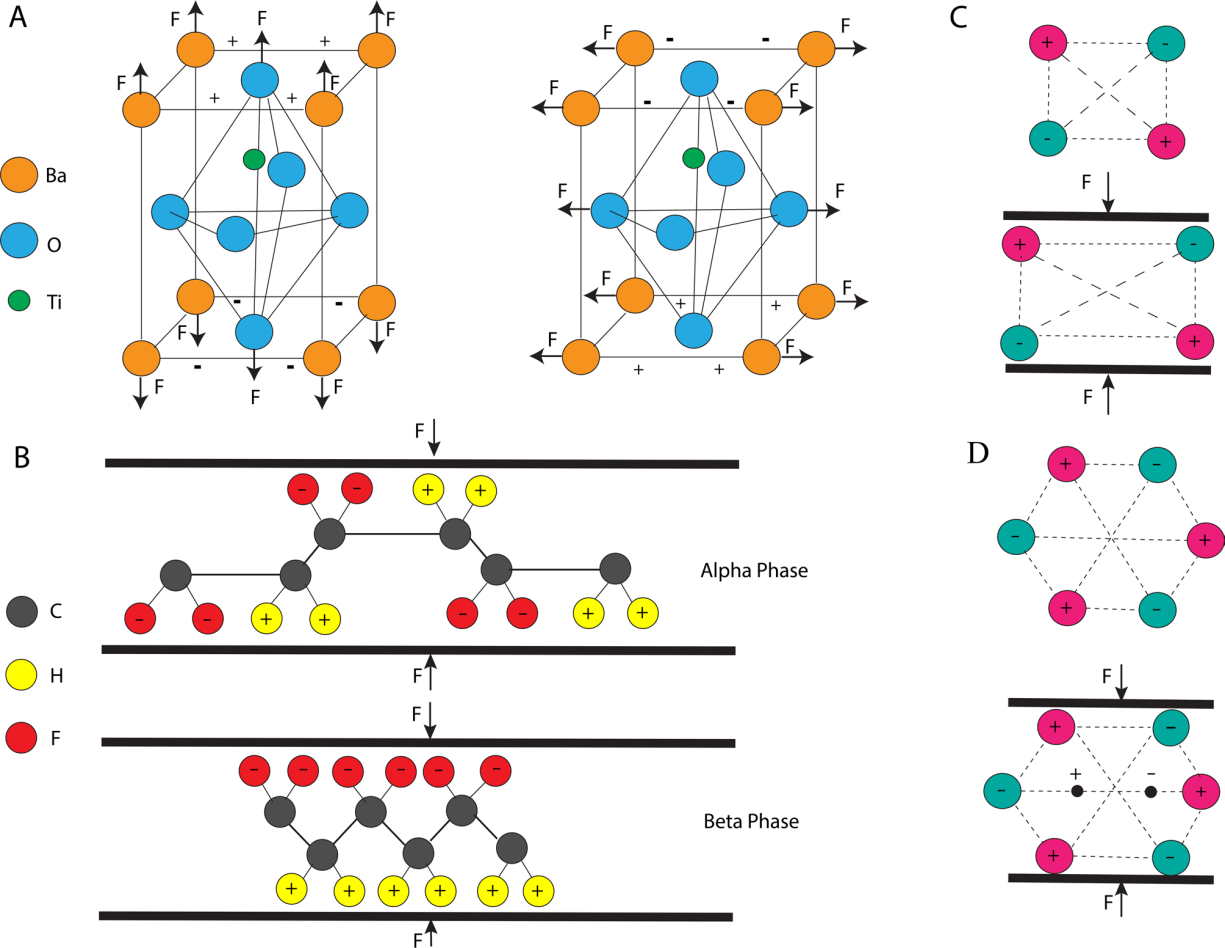
(**A**) Tetragonal crystal structure of barium titanate. Piezoelectricity arises from the off-center displacement of the Ti⁴⁺ ion within the oxygen octahedron. In the unstressed tetragonal phase, Ba²⁺ (orange), Ti⁴⁺ (green), and O²⁻ (blue) ions occupy positions that generate spontaneous polarization due to the Ti⁴⁺ cation being displaced relative to the surrounding oxygen octahedron. When mechanical force (F) is applied, the oxygen and barium ions shift relative to the titanium ion, altering the unit-cell dipole moment. This stress-induced change in polarization generates an electric signal, demonstrating the piezoelectric response of BaTiO₃. The two subfigures illustrate that both the direction and magnitude of polarization change depend on the direction of the applied mechanical stress. **(B)** α and β phases of PVDF. In the α-phase, the polymer chain adopts a TGTG′ conformation in which dipole moments from the C–F and C–H bonds alternate in direction, resulting in mutual cancellation. When stress is applied, this symmetric arrangement prevents the generation of a net electrical output, rendering the α-phase non-piezoelectric. In contrast, the β-phase has an all-trans configuration that aligns all C–F dipoles in the same direction. Under applied stress, these aligned dipoles shift collectively, producing a change in polarization and a measurable piezoelectric signal. **(C)** A centrosymmetric crystal with symmetrically arranged positive and negative charge centers. Due to the presence of a center of inversion, dipole moments from oppositely positioned ions cancel, yielding zero net polarization. When mechanical force is applied, ionic displacements remain symmetric with respect to the inversion center, preventing the development of charge asymmetry. Consequently, the material cannot exhibit piezoelectricity. **(D)** A non-centrosymmetric lattice lacking inversion symmetry, enabling a spontaneous dipole moment. Upon application of mechanical stress, ionic positions shift in a direction-dependent manner, altering the separation between charge centers and generating a net change in polarization, which enables a piezoelectric response. Images C) and D) reproduced with permission from [Ramadan *et al.*], “[A review of piezoelectric polymers as functional materials for electromechanical transducers],” [Smart Materials and Structures], © [2014], IOP Publishing

At the heart of piezoelectricity is a non-centrosymmetric crystal lattice (or oriented polymer structure) in which mechanical strain causes a shift in charge distribution [66]​. Common inorganic piezoelectric materials include crystals or ceramics like quartz (SiO₂), barium titanate (BaTiO₃), and lead zirconate titanate (PZT). There are also piezoelectric polymers such as poly(vinylidene fluoride) (PVDF) and its copolymers, which contain aligned molecular dipoles that produce a charge when stretched​ [67]. Figure 3 illustrates the concept of piezoelectricity, showing how mechanical stress applied to piezoelectric materials generates an electric charge. Nanoscale forms of these materials (nanoparticles, nanowires, nanotubes) retain piezoelectric properties. For instance, barium titanate nanoparticles (BTNPs) have been widely studied as ultrasonic neural stimulators due to their strong piezoelectric response and biocompatibility​​. When ultrasound vibrates a BTNP, it generates an alternating voltage that can depolarize neurons and trigger action potentials if above threshold [68].

A key advantage of piezoelectric nanostimulators is that they directly interface with the bioelectric excitability of neurons. Neuronal membranes are essentially electrical capacitors, and small changes in the local electric field can open voltage-gated ion channels. By planting piezoelectric nanoparticles in or near target neurons, one can deliver electrical stimuli to those cells with exquisite spatial specificity (limited to where the nanoparticles reside) and temporally gated by the ultrasound pulses. This could achieve neuron-level stimulation without needing implanted macroelectrodes and with minimal spread to neighboring cells that lack nanoparticles.

#### Applications of piezoelectric nanosystems for neuromodulation

##### *In vitro* neuromodulation with piezoelectric nanosystems

As we discussed in the previous section how piezoelectric materials can generate electrical signals and stimulate neurons. This has been successfully demonstrated in several studies. For instance, Marino *et al.* showed that barium titanate (BaTiO₃) nanoparticles introduced to neuron-like cells (SH-SY5Y) enabled ultrasonic activation of those cells’ calcium signalling​​. In their experiment, cells exposed to 0.8 W/cm² pulses at 1 MHz showed large calcium spikes only when BTNPs were present; ultrasound alone at the same intensity produced minimal response. Pharmacological tests indicated that the response was mediated by normal voltage-gated ion channels (Na⁺ and Ca²⁺ channels), not by nonspecific mechanosensitive channels, implying that the piezoelectric effect was effectively depolarizing the cells to trigger action potentials​​. This study was important as it ruled out mere mechanical or thermal artifacts and confirmed a genuine bioelectric stimulation via the nanoparticles [69].

Follow-up work by Genchi *et al.* took a hybrid materials approach: they created composite films of PVDF-TrFE polymer embedded with BTNPs and seeded neurons on these films​​. When ultrasound was applied, only neurons on piezoelectric substrates (PVDF or PVDF+BTNP) showed induced calcium transients, whereas those on non-piezoelectric controls did not​. Moreover, chronic, repeated ultrasound stimulation over days led to enhanced neuronal differentiation on the piezoelectric films – neurons exhibited longer neurites and a higher percentage of β3-tubulin-positive cells (a neuronal marker), indicating that the electrical stimulation from the material promoted growth and maturation​​. This suggests a potential regenerative or therapeutic angle: in addition to acute stimulation, piezo-nanostimulation could encourage neuroplasticity or neurogenesis over time [70].

Other studies have explored different piezoelectric nanomaterials. Boron nitride nanotubes (BNNTs) are one such material – they are piezoelectric and have been reported to enhance neurite outgrowth under ultrasound in PC12 neuronal cells​ [71]. Additionally, new composite constructs like magneto-piezoelectric microdiscs have been tested, which allow magnetic targeting of the nanoparticles to a location, then ultrasound to stimulate them electrically​ [72]. The combination of modalities (e.g. magnetic guidance + ultrasonic activation) could further refine how we deliver and use these nanotransducers *in vivo*.

##### *In vivo* demonstrations

One of the milestones for piezoelectric neuromodulation was achieving effects in living animals. Chen *et al.* (2022) developed carbon-coated BTNPs and showed that when injected into a rodent brain, ultrasound could elicit neuronal activation evidenced by calcium imaging and upregulation of activity markers like c-Fos​ [73]. Even more striking, they reported improvements in a dopamine-related behavior in a zebrafish model when applying ultrasound with these nanoparticles, hinting at therapeutic potential for disorders like Parkinson’s​ [74]. In another study, Yoo *et al.* (2022) used biodegradable piezoelectric nanogenerators implanted near a peripheral nerve injury site; ultrasound activation of these generators promoted nerve tissue repair by electrically stimulating regenerating neurons​ [75]. This suggests that beyond central brain stimulation, piezoelectric effects can aid in neurorehabilitation contexts. A recent advancement is the development a fully-implantable, flexible piezoelectric micromachined ultrasound transducer (pMUT) device, termed “ImPULS,” which is a fully-encapsulated, flexible piezoelectric micromachined ultrasound transducer that incorporates a biocompatible piezoceramic, potassium sodium niobate [(K, Na)NbO_3_]. In *ex vivo* hippocampal slices, ImPULS excited neurons which was confirmed by via GCaMP7F calcium imaging). *In vivo* implantation studies in mice further showed an increase in c-Fos expression when the dorsal CA1 region was stimulated, while targeted stimulation within the substantia nigra pars compacta (SNc) elicited time-locked elevations in striatal dopamine release, as detected using a GRAB-DA2m fluorescent sensor. Importantly, no activation was observed when stimulation was applied approximately 200 μm from the SNc, underscoring the high spatial specificity of this approach. Despite its promise, the current ImPULS architecture exhibits several technical constraints. The acoustic output is limited to peak pressures of approximately 100 kPa due to the polarization saturation limits of the piezoceramic thin film. Moreover, the long-term biointerface stability and optimization of stimulation parameters such as frequency, duty cycle, and pulse repetition frequency remain to be studied for achieving cell-type-selective and reproducible neuromodulation *in vivo* [76]. In another study Kim *et al.* developed an “implantable ultrasound stimulator” consisting of an array of piezoelectric microelectromechanical system (MEMS) transducers that can be placed on the skull or dura to deliver localized ultrasound to piezoelectric nanoparticles distributed in the brain. They fabricated a one-dimensional piezoelectric micromachined ultrasound transducer (pMUT) array (34 channels, 60 μm diameter membrane elements) which was characterized to produce focused acoustic pressures of ~ 0.5 MPa at 5 mm distance. Using human neuroblastoma SH-SY5Y cells as a model, ultrasound exposure (9 MHz, 15 Vpp, PRF = 1 kHz) for durations of 10, 20 and 40 min significantly increased neurite outgrowth (mean lengths: ≈ 90.9 ± 19.2 μm, 108.5 ± 28.4 μm, 119.9 ± 34.3 μm respectively) compared to non-stimulated controls (63.2 ± 17.3 μm). They further developed magnetically actuated “Cellbots” (SH-SY5Y cells loaded with PLL-coated superparamagnetic iron oxide nanoparticle clusters), which were guided to a target region via a rotating magnetic field (20 mT, 10 Hz) and then locally stimulated with the pMUT array. In the targeted region, stimulated Cellbots exhibited neurite lengths of ≈ 116.7 ± 39.2 μm compared to ≈ 57.2 ± 11.3 μm in non-stimulated regions, confirming spatially selective differentiation [77]. The ability to pattern neural network formation *in vivo* using this method could open new frontiers in brain repair. Recently Tang *et al.* (2025) developed ultrasound-activated piezoelectric nanostimulators (PNSs) based on barium titanate@polydopamine (BTO@PDA) nanoparticles for minimally invasive, long‐acting wireless deep brain electrical stimulation to suppress epileptic seizures. The BTO@PDA nanostimulators (average diameter ≈ 180 nm, piezoelectric coefficient d₃₃ = 190 pC N⁻¹) generated electrical potentials of 0.2–1.0 mV under low‐intensity ultrasound (I_SPPA_ = 0.3–0.6 W cm⁻², 1 MHz) and adhered stably to neuronal membranes without cellular internalization. *In vitro*, they preserved > 90% neuronal viability after 72 h at 200 µg mL⁻¹ and effectively activated neurons via voltage‐gated calcium channels, as confirmed by Fluo-4 AM imaging and c-Fos upregulation. When stereotactically implanted into deep brain regions (e.g., hippocampal CA1 or VTA), the nanostimulators remained positionally stable for at least 14 weeks with minimal microglial activation compared to conventional deep brain stimulation (DBS) electrodes. Under ultrasound stimulation (1 MHz, PRF = 80 Hz, duty cycle 10%), implanted PNSs activated local neurons and the VTA–NAc circuit in rats, producing intensity-dependent calcium responses without detectable thermal rise (< 0.6 °C). In optogenetic epilepsy models, ultrasound-activated PNSs increased seizure latency from ≈ 53 s to 120 s and reduced the maximum EEG variance by ≈ 3-fold, while in pilocarpine-induced acute epilepsy, latency to first generalized seizure increased from ≈ 30 min to 65 min. In chronic epilepsy models, daily ultrasound (0.45 W cm⁻², 5 Hz) reduced spontaneous seizure frequency by 2–3 events per day and improved cognitive and anxiety-related behaviors. Overall, the study demonstrates that low-intensity ultrasound combined with BTO@PDA nanostimulators enables safe, stable, and effective deep brain neuromodulation, offering a quantitative, non-invasive alternative to implanted DBS systems for epilepsy therapy [78].

In summary, piezoelectric nanosystems have shown robust capability to stimulate neuronal activity when triggered by US. They effectively bridge ultrasound and the excitable membrane, acting as nanoelectrodes. The groundwork in cell models has progressed to animal studies, demonstrating not only acute neuromodulation (e.g. inducing movements or neural signals) but also longer-term neuromodulatory therapies (e.g. enhancing regeneration). Future challenges include ensuring these nanoparticles can be delivered to specific brain regions (potentially via targeted delivery vectors or focused BBB opening) and determining their fate and safety profile after repeated use. There is also ongoing work to optimize their efficiency so that even lower ultrasound intensities (fully within diagnostic safety limits) can be used to drive neural activity, which would be important for human applications. A summary of studies employing ultrasound-assisted piezoelectric nanosystems for neuromodulation and therapeutic applications is presented in Table 4.

**Table 4:** Summary of studies employing ultrasound-assisted piezoelectric nanosystems for neuromodulation and therapeutic applications

Reference	Nanosystem	Ultrasound parameters	*In vitro/In vivo*	Animal model/ cell type	*In vivo *target	Neuromodulatory effects	Therapeutic effects
Li *et al.* (2025) [168]	Hafnium-based metal–organic framework (UIO) nanoparticle coated with BV2 cell membranes functionalized with transferrin receptor (TfR)-targeting peptides (UIO-S@BMT) encapsulated with carbamazepine D = 180 nm	*f =* 1 MHz, *I =* 1.0 Wcm^− 2^, DC = 50%, *t =* 5 min	*In vivo*	*In vitro:* SH-SY5Y, bEnd.3, BV2 and MA-c *In vivo:* Kainic acid induced epileptic model in Sprague–Dawley rats	Intravenous injection via tail vein	Partial neuroprotection by restoring 35–40% of NeuN-positive cells.	• Suppression of abnormal electrophysiological discharges. • Significant seizure attenuation with Racine scores reduced to 1.0 ± 0.8 (mild or minimal seizure activity). • Near complete normalization of SEEG waveforms and time-frequency spectral patterns. • Shortened seizure duration to 407.1 ± 36.4s (60.5% reduction relative to the model group). • 77.2% of normal locomotor activity restored.
Tang *et al.* (2025) [78]	BaTiO_3_ (coated with polydopamine) D = 180 nm	*f =* 1 MHz, PRF = 80 Hz, DC = 10%, I_SPPA =_ 0.45 Wcm^− 2^ *t* = 3 min	Both	*In vitro*: LUHMES-Derived Neuronal Culture *In vivo*: Male Wistar rats	Left ventral tegmental area (VTA) and Hippocampal CA1 region via intracranial injection	*In vitro*: • Increase in intracellular Ca^2+^ signal. • c-Fos expression increased by ≈ 1.3-fold compared to uncoated BaTiO_3_. *In vivo*: • Increase number of c-Fos positive cells around the injection site. • Rapid, reversible increase in calcium fluorescence in neurons which increased with higher US intensities. • Neuromodulatory effects remained stable up to 16 weeks post-injection.	• Significant suppression of seizures. • Maximum value of variance and mean absolute deviation of EEG during light irradiation decreased by 1.68 and 2.93-fold respectively. • US delayed onset of EEG changes by ≈ 26 min. • Latency to the first generalized seizure prolonged from 29.8–65.2 min, however, no effect on seizure onset score. • Significant reduction in total seizure onset times, with 2–3 fewer seizures than in control. • Improved locomotor activity, reduced anxiety-like behaviors, and partially restored cognitive and memory function in treated rats.
Zhang *et al.* (2024) [169]	(K, Na)NbO_3_) (KNN) NPs (coated with cholesterol - KNNC) D = 319 nm	*f =* 1.5 MHz, PRF = 100 Hz and 1 kHz, *P* = 0.05–0.2 MPa	*In vitro*	• Human neuroblastoma cells (SH-SY5Y) • Neural stem cells from rat embryo	NA	• Ca^2+^ transients increased with increase in acoustic pressure. • Significant enhancement of neural stem cell (NSC) differentiation into mature neurons (↑Tuj1, ↑MAP2, ↓GFAP), synapse formation (↑SYN1/PSD95 puncta), and Ca²⁺ influx-driven neuromodulation. • Upregulation of CaMKII expression, suggesting improved synaptic plasticity via Ca²⁺/CaM signaling.	NA
Chen* et al.* (2024) [170]	PEP@BT D = 45–75 nm	*f =* 1 MHz, *I* = 1.5 W/cm^2^, *t =* 5 min	Both	*In vitro:* • Rat neuronal like cells (PC-12) *In vivo:* • 6-hydroxydopamine induced Parkinson’s disease model in zebrafish • PD model C57BL/6 mice	• Midbrain via intracranial injection in the zebrafish • Right Striatum of the mice via intracranial injection	*In vitro:* • 60% increase in intracellular Ca^2+^ signal. • 2.5-fold increase in TH expression after 5-day exposure to US. *In vivo:* • Expression of TH restored in substantia nigra of the mice.	• Restoration in swimming activities of the zebrafish including an increase in the total distance moved, decrease in rest time and a higher percentage of total activity in the whole zone. • In mice the average movement speed increased, the duration of passing a specific distance reduced and the movement disorders of mice were significantly improved.
Zheng *et al.* (2024) [171]	Tetragonal BaTiO_3_ NPs (coated with PEG) D = 459.16 ± 130.98 nm	*f =* 1 MHz, *I* = 0.5 W/cm^2^, DC = 50%, *t =* 10s	*In vitro*	Primary neurons from fetal rats	NA	• Significant decrease in basal Ca^2+^ observed in epileptic neurons. • Increase in cell viability.	NA
Han* et al.* (2024) [172]	Piezoelectric magnetic Janus microparticles (PEMPs) Porous silica microparticles (20 μm) conjugated with BaTiO_3_ NPs (20 nm) on half surface and Ni/Au thin film on the other half	*f* = 2 MHz, pulse duration = 100ms, *I* = 5–100 mW/cm^2^, Burst Frequency = 0.5–200 Hz, *t* = 20 min	*In vitro*	Primary rat hippocampal neurons	NA	• FUS pulses of 50 W/cm^2^ increased the spiking frequency up to 5 times. • Single PEMP could stimulate neurons > 60%, up to 40 μm distance.	NA
Fan *et al.* (2023) [173]	Molybdenum Disulfide Nanosheets (MoS_2_ NS) D = 543.9 ± 90.9	*f =* 2 MHz, *P* = 400 kPa, pulse duration = 500ms, cycles = 1,000,000	Both	*In vitro:* Human neuroblastoma cells (SH-SY5Y) *In vivo:* Wildtype C57BL/6J mice	Septal nucleus region via intracranial injection	*In vitro:* Increase in Ca^2^ flux by 37.9 ± 7.4%. *In vivo:* Increase in c-Fos expression by 3-fold.	NA
Chen *et al*. (2023) [73]	(PVDF/TMCM-MnCl_3_)	*f* = 100 kHz. *I* = 25 mW/cm^2^, pulse duration = 60µs, delay = 10ms, *t* = 30 min	*In vivo*	Parkinson’s disease model in Sprague-Dawley (SD) rats	Subthalamic nucleus (STN)	Modulation of aberrant β oscillations.	• Preservation of tyrosine hydroxylase (TH)-positive neurons in the substantia nigra and striatum. • Increase in average velocity and duration of locomotion in rats.
Genchi *et al.* (2022) [70]	P(VDF-TrFE)/BaTiO_3_ D = 300 nm tetragonal crystals in P(VDF-TrFE) film	*I* = 1 W/cm^2^, Burst Rate = 100 Hz, 5s twice a day for 6 days	*In vitro*	Human neuroblastoma cells (SH-SY5Y)	NA	• Increase in amplitude of Ca^2+^ transients. • Increase in β3-tubulin positive cells. • Increase in neurite length under chronic stimulation.	NA
Kim *et al.* 2022 [174]	*N*,*N*′-di-*sec*-butyl-*N*,*N*′-dinitroso- 1,4-phenylenediamine (BNN6) and piezoelectric barium titanate nanoparticle (BTNP) coated with polydopamine (pDA) D = 261.15 ± 5.14 nm	*In vitro:* *f =* 1 MHz, pulse duration = 10ms, DC = 50%, PRF = 10 Hz *In vivo:* *f =* 1.5 MHz, *I* = 462.4 W/cm^2^, DC = 10%, PRF = 10 Hz, *t* = 60s for 10 days	Both	*In vitro:* Human neuroblastoma cells (SH-SY5Y) *In vivo:* Acute Parkinson’s disease model in C57BL/6 mice	Intravenous injection of nanoparticles	*In vitro:* Increase in intracellular Ca^2+^ concentration. • Significant increase in dopamine concentration in the media. *In vivo:* Significant increase in c-Fos positive cells of the subthalamic nucleus (STN). • Increase in Tyrosine hydroxylase (TH) positive neurons.	• Gradual improvement • in motor functions along with daily sequential stimulation for 10 days in rotarod test. • Locomotor activity recovered on day 16.
Zhao *et al.* (2020) [74]	BaTiO_3_ nanoparticles (BTNPs) with carbon shell – C@BT D = 66 ± 10 nm	P_w_ = 120 W, stimulation on day 1, 5 and 7 post nanoparticle injection.	*In vivo*	6-hydroxydopamine induced Parkinson’s disease model in zebrafish.	Midbrain via intracranial injection.	Tyrosine hydroxylase levels normalized after EMF exposure.	Increase in movement in open field tests, flexible motor behaviour and higher percentage of total activity in center zone of the open field.
Liu *et al.*(2020) [175]	*S.platensis*@ Fe_3_O_4_ @tBaTiO_3_ L = 22 μm, W = 5 μm, D = 0.6 μm Fe_3_O_4_: D = 13 ± 3 nm tBaTiO_3_: D = 58 ± 15 nm (coated with gum Arabic)	*f* = 1 MHz, *I* = 1 W/cm^2^, *P* = 1 MPa, *t* = 5s, three times a day for 3 days	*In vitro*	Rat neuronal like cells (PC-12)	NA	• Increase in amplitude of Ca^2+^ transients by 196%. • Differentiation with an increase in neurite outgrowth.	NA
Chen *et al.* (2019) [176]	BaTiO_3_ D = 100 nm (coated with DSPE-PEG-5000	*f =* 500 kHz, *P* = 2 kPa, *t* = 10s	*In vitro*	Primary rat cortex neurons	NA	Increase in spike numbers and calcium transients.	NA
Rojas *et al.* (2018) [177]	Barium Titanate Nanoparticles (BTNPs) D = 116 ± 46.5 nm (coated with gum Arabic	*f* = 1.5 MHz, *I* = 1 W/cm^2^, pulse duration = 2s, *t* = 180s, DC = 50%	*In vitro*	Primary neurons from hippocampus and cortex from rat embryo.	NA	Increase in neuronal firing rate correlated to the US pulses.	NA
Marino *et al.* (2015) [69]	Tetragonal barium titanate nanoparticles (BTNPs) D = 479 ± 145.3 nm (coated with gum Arabic)	*f* = 1 MHz, *I* = 0.1–0.8 W/cm^2^, t = 5s	*In vitro*	Human neuroblastoma cells (SH-SY5Y)	NA	• Increase in amplitude of Ca^2+^ transients at higher intensities. • Highest amplitude of Ca^2+^ transient at 0.8 W/cm^2^.	NA
Ciofani *et al.* (2010) [71]	Boron nitride nanotube (BNNTs) L = 200–600 nm, D = 50 nm (coated with glycol-chitosan)	*f* = 40 kHz, *P*_*w*_ = 20 W, *t* = 5s, four times a day for 9 days.	*In vitro*	Rat neuronal like cells (PC-12)	NA	• Increase in neuronal processes per differentiated cell. • Increase in developed neurites.	NA

* *f* = Center frequency, PRF = Pulse repetition frequency, *P* = Acoustic Pressure, *t* = time duration, P_w_ = Power, D = Diameter, W = Width, L = Length, NP = Nanoparticles, US = Ultrasound, *I* = Intensity, PEP = Piezoelectric polymer, BT = Barium Titanate, TH = Tyrosine Hydroxylase, PD = Parkinson’s disease, PEG = Polyethylene glycol, DSPE = 1,2-Distearoyl-sn-glycero-3-phosphoethanolamine, EMF = Electromagnetic force

#### Materials and design considerations

For effective neuromodulation, piezoelectric nanoparticles should produce sufficient field strength at the neuronal membrane. The yield depends on the material’s piezoelectric charge coefficient *d* (how much charge per unit force)​, the particle size, and the ultrasound pressure [65, 79]. Typical BTNPs (50–500 nm diameter) can emit electric potentials of several millivolts under moderate ultrasound​​. While that might seem small, clustering of particles or resonant enhancements can increase the effect. Additionally, placing nanoparticles on the neuronal membrane (e.g. by functionalization to stick to cell membranes) maximizes the coupling of the field to the cell [80]. Novel composites, such as piezoelectric polymer matrices embedded with ceramic nanoparticles, have also been created to boost piezoelectric output and provide flexible substrates for cell growth. For example, a film of P(VDF-TrFE) polymer with BTNP fillers can deform under ultrasound and charge the surface, effectively stimulating cells cultured on it [70]​​. Biocompatibility is another consideration. Many piezoelectric ceramics contain lead (PZT), which is undesirable for *in vivo* use. Thus, lead-free alternatives like barium titanate, zinc oxide, or certain perovskite oxides are preferred for medical applications. Coatings (e.g. silica or polymer coatings) can also be applied to nanoparticles to improve stability and reduce any toxicity or aggregation in biological fluids.

### Gas vesicles and microbubbles for mechanical transduction

#### Working principle

Gas-filled nanostructures are extremely effective at converting ultrasound into mechanical work. Even a low-intensity ultrasound field will cause a gas bubble to oscillate in size (expand and contract) due to pressure changes [81–84]. These oscillating microbubbles push and pull on nearby cells, generating microstreaming in fluids and mechanical stresses on cell membranes. In the context of neuromodulation, microbubble oscillation can activate mechanosensitive ion channels in neurons, leading to ionic currents and neuronal firing​​ [85]. Among these channels, the transient receptor potential (TRP) channels and the K2P family, comprising six subfamilies, including TWIK and TREK, play crucial roles in mechanotransduction. Key members of the K2P family, such as TREK-1, TREK-2, and TRAAK, are predominantly expressed in sensory neurons and exhibit sensitivity to diverse mechanical stimuli [47]. The Piezo family constitutes another significant group of MS ion channels, playing critical roles in mechanosensory processes, development, and regulatory functions [48]. If driven harder, bubbles can transiently collapse (inertial cavitation), producing shockwaves and jets that can porate cell membranes or disrupt the BBB [35]. For neuromodulation purposes, typically stable oscillation (stable cavitation) is utilized at controlled acoustic pressures to avoid tissue damage while still providing a mechanical stimulus.

Gas vesicles (GVs) are a particular class of nanoscale gas-filled organelles originally derived from bacteria and archaea. They are protein-shelled nanobubbles (typically 100–300 nm in length) that are air-filled and stable in aqueous media​​ [86, 87]. GVs can be genetically engineered or produced from bacterial cultures, and they have been proposed as “acoustic actuators” in biotechnology​ [37]. When exposed to ultrasound, gas vesicles can oscillate or even rupture, acting as localized sources of mechanical force. Recent studies have shown that introducing GVs to neurons greatly lowers the ultrasound intensity needed to achieve neuromodulation​. For example, Hou *et al.* demonstrated that 250 kHz pulses at only 0.2 MPa could reliably trigger calcium influx in neurons *in vitro* when GVs were present, whereas the same ultrasound without GVs had little effect​. The GVs effectively serve as nano-transducers that amplify the acoustic energy at the cellular level, bending neuronal membranes or tugging on mechanically gated channels such as Piezo1 or TREK-1 [37].

A compelling feature of gas vesicles is that they can be genetically targeted [88, 89]. Researchers have developed acoustic reporter genes that cause specific cells to produce gas vesicles, thereby making those cells ultrasound-responsive [90]​. Although this involves genetic modification (similar in principle to optogenetics), it offers a noninvasive means to “install” bubble-like actuators in chosen cell types [37]. Another approach is to deliver GVs via the bloodstream to a target region; because ultrasound can be focused, only bubbles within the focal spot will oscillate significantly, confining the effect to the sonicated area [91]. Gas vesicles, being nanoscale and biogenic, offer a way to get gas inclusions into the extravascular space or even inside cells. For instance, GVs are inherently smaller and more stable than typical microbubbles, making them attractive for repeatable neuromodulation. One trade-off is that GVs can collapse at higher pressures (around 0.5–1 MPa) [92]. This can be harnessed for one-time effects like BBB opening​ or drug release, but for sustained neuromodulation, GVs must be operated below the rupture threshold so that they can oscillate non-destructively.

Ultrasound microbubble contrast agents (like lipid-shelled microbubbles ~ 1–5 μm in diameter) have also been used to assist neuromodulation. Microbubbles injected intravenously can cross into the brain’s vasculature and augment the effects of a transcranial ultrasound pulse [93]. Studies show that ultrasound combined with microbubbles can stimulate motor responses at lower acoustic pressures than ultrasound alone [94]​​. The microbubbles magnify radiation forces and produce acoustic micro-streaming that likely influences nearby neurons or mechanoreceptors​ [92].

In summary, gas vesicles and other bubble-based nanosystems leverage mechanotransduction by converting US into mechanical stimuli that engage stretch-sensitive neuronal pathways. They are particularly promising for neuromodulation because of the strong effect even at safe ultrasound intensities, they can produce significant mechanical perturbations at the cellular scale​. Moreover, as discussed later, bubble systems can serve a dual role by also facilitating drug delivery when driven at higher intensities to momentarily open the BBB or to release drug payloads.

#### Applications of gas vesicles for neuromodulation

Gas vesicle-aided neuromodulation has advanced rapidly since the initial reports a few years ago. As discussed earlier, GVs act as local amplifiers of acoustic pressure, converting ultrasound into mechanical stimuli that neurons can detect​. Two notable studies by Hou* et al.* in 2021 and 2024 have demonstrated the potential of this approach:

In Hou *et al.* (2021)​, primary cortical neurons were incubated with gas vesicles (from *Anaerobacterium* species) and then stimulated with low-intensity FUS. The presence of GVs made the neurons highly responsive to ultrasound, showing repeatable calcium spikes precisely time-locked to ultrasound pulses (whereas control neurons without GVs showed little or no response)​. The neurons also upregulated c-Fos, confirming genuine activation rather than just calcium influx. Mechanistically, blocking mechanosensitive ion channels significantly reduced the GV-enhanced responses, indicating that stretch-activated channels (likely Piezo or TRP family) were involved in transducing the mechanical effects to electrical signals​. Importantly, the study found no sign of membrane damage or cytotoxicity from the GV-mediated stimulation – neurons remained viable, and no uptake of membrane-impermeant dyes was seen, suggesting that the mechanical stimuli were below injurious levels​ [37].

Hou *et al.* (2024) extended this to *in vivo* neuromodulation in mouse brain​. They injected GVs into specific brain regions (like the motor cortex or deep nuclei) via a micro-cannula and showed that FUS could then activate those regions with unprecedented spatial precision​. By delivering GVs to one side of the motor cortex, ultrasound stimulation evoked movements (EMG activity in contralateral limbs) only when targeting that side; moving the focus even a millimeter away (where no GVs were present) failed to produce an effect​. Furthermore, by injecting GVs into two nearby deep brain areas (e.g. two subregions of the basal ganglia), the authors could use ultrasound to selectively trigger distinct behaviors: one target produced freezing (a fear response), the other produced rotational movement, depending on which spot was sonicated​. This level of selectivity, essentially addressing two different neural circuits just millimeters apart by choosing where to put nanobubbles, is extremely difficult with any other noninvasive method. Calcium imaging *in vivo* confirmed that only neurons in the vicinity of GVs showed calcium transients during FUS, and c-Fos mapping showed activation confined to sub-millimeter regions containing GVs​. They also found that ultrasound neuromodulation via GVs could alleviate a pathological behavior: FUS stimulation of GVs in a depression model (inducing activity in a mood-related circuit) reduced depression-like behavior in mice [95]​. This hints at therapeutic uses of the technique. Notably, these effects were achieved with low ultrasound pressures (~ 0.08–0.3 MPa) that by themselves would likely have no neuromodulatory impact​. The GVs lowered the required dose of ultrasound and made the stimulation repeatable and reversible, as neurons would consistently fire with each burst and then return to baseline shortly after​.

Parallel to these, other researchers have used synthetic microbubbles or nanodroplets in the bloodstream to enhance FUS neuromodulation. For example, Cui *et al.* (2020) demonstrated that transcranial FUS in rats produced significantly more motor responses when microbubbles were present, especially at lower acoustic intensities that normally would be sub-threshold [92]​. The microbubbles boosted the mechanical effect, essentially acting similarly to gas vesicles but confined to capillaries. The risk with microbubbles is unwanted BBB opening if the pressure is too high​; however, when carefully calibrated, one can keep BBB intact and still get neuromodulatory benefits.

#### Biosafety and Engineering of GVs

One concern for using bacterial gas vesicles *in vivo* is the immune reaction. GVs are protein shells and could be seen as foreign by the immune system. A recent study showed that PEGylating the surface of gas vesicles reduced their immunogenicity significantly, allowing repeated dosing with less clearance by macrophages​ [96]. Additionally, “humanizing” the proteins via synthetic biology or using human-derived sequences might further reduce immune responses in future designs. On the other hand, GVs have an advantage of being eventually biodegradable (since they are protein-based) and not leaving toxic remnants. In the studies so far, no overt toxic effects were observed; histology from Hou *et al.* (2021) showed no tissue damage or apoptosis from GV-mediated ultrasound, and behaviorally the animals tolerated the injections and sonications well​ [37].

In conclusion, gas vesicle-based neuromodulation has rapidly progressed to enable noninvasive, deep, and cell-type-neutral stimulation with spatial precision below 1 mm​. By providing a means to “acoustically label” specific brain locations or cell populations, it represents a paradigm shift in how focused energy can interface with neural circuits. Future work will likely explore targeted delivery of GVs (perhaps using cell-specific antibodies or gene delivery of acoustic reporter genes) so that even cell-type-specific neuromodulation might be achieved with ultrasound. Another avenue is using GVs not just for stimulation but for inhibition e.g. activating mechanosensitive K⁺ channels to hyperpolarize cells. Given that mechanosensitive channels like TREK-1 are inhibitory, one could conceive of tuning the ultrasound parameters or vesicle properties to bias towards neuronal inhibition instead of excitation, offering a way to turn off overactive cells (useful in epilepsy, for instance). Overall, GVs have established themselves as one of the most promising tools in the FUS neuromodulation arsenal. A summary of studies employing ultrasound-assisted gas vesicles for neuromodulation and therapeutic applications is presented in Table 5.

**Table 5:** Summary of studies employing ultrasound-assisted gas vesicles for neuromodulation and therapeutic applications

Reference	Nanosystem	Ultrasound parameters	*In vitro/in vivo*	Animal model/ cell type	*In Vivo* target	Neuromodulatory effects	Therapeutic effects
Hou* et al.* (2024) [95]	Nano gas vesicles from *Anabaena flos-aquae* (coated with PEG) W = 50–100 nm L = 200–600 nm	*In vitro:* *f* = 1 MHz, *P* = 0.20 MPa, 300-tone burst pulses, delay = 10s, DC = 10%, PRF = 1 kHz *In vivo:* *f* = 1 MHz, *P* = 0.17–0.54 MPa, DC = 40%	Both	*In vitro:* Primary cortical neurons from rat embryos. *In vivo:* C57BL/6 mice	Dorsal striatum and Dorsal raphe nucleus (DRN)	*In vitro:* Increase in amplitude of Ca^2+^ transients (increase in fluorescence intensity by 46.2%) *In vivo:* Elevated c-Fos in the brain area injected with gas vesicles.	• Neural activation in the motor cortex and striatum, eliciting precise behavioral responses including limb movement, rotation (3 ± 0.5 mm), gait freezing and 18-fold growth in linear speed. • Activation of serotonergic neurons in DRN increased 5-HT release. • Reduction in depression-like behaviors in mice.
Hou* et al.* (2021) [37]	Gas Vesicles from *Anabaena flos-aquae* W = 50–100 nm L = 100–500 nm	*In vitro:* *f* = 1 MHz, *P* = 0.20 MPa, pulse duration = 300 ms (5 pulses), DC = 10%, PRF = 1 kHz *In vivo:* *f* = 1 MHz, *P =* 0.08 MPa, pulse width = 300µs, pulse duration = 300ms, *t* = 40 min	Both	*In vitro:* Mouse hippocampal cell line mHippoE-18 (CLU199) and Primary rat cortical neurons. *In vivo:* Male C57BL/6 rats (expressed with GCaMP6s through viral transduction)	Ventral tegmental area (VTA)	*In vitro:* • Stable and reversible Ca^2+^ transients neurons were able to recover after each pulse. *In vivo:* • Repeated, rapid, and consistent increase in GCaMP6s fluorescence. • Higher number of c-Fospositive cells in VTA.	NA

* *f* = Center frequency, PRF = Pulse repetition frequency, *P* = Acoustic Pressure, *t* = time duration, P_w_ = Power, D = Diameter, W = Width, L = Length, NP = Nanoparticles, US = Ultrasound, *I* = Intensity, 5-HT = 5-hydroxytryptamine, VTA = Ventral Tegmental Area

### Mechanoluminescent nanosystems for sono-optogenetics

#### Working principle

**Fig. 4:**
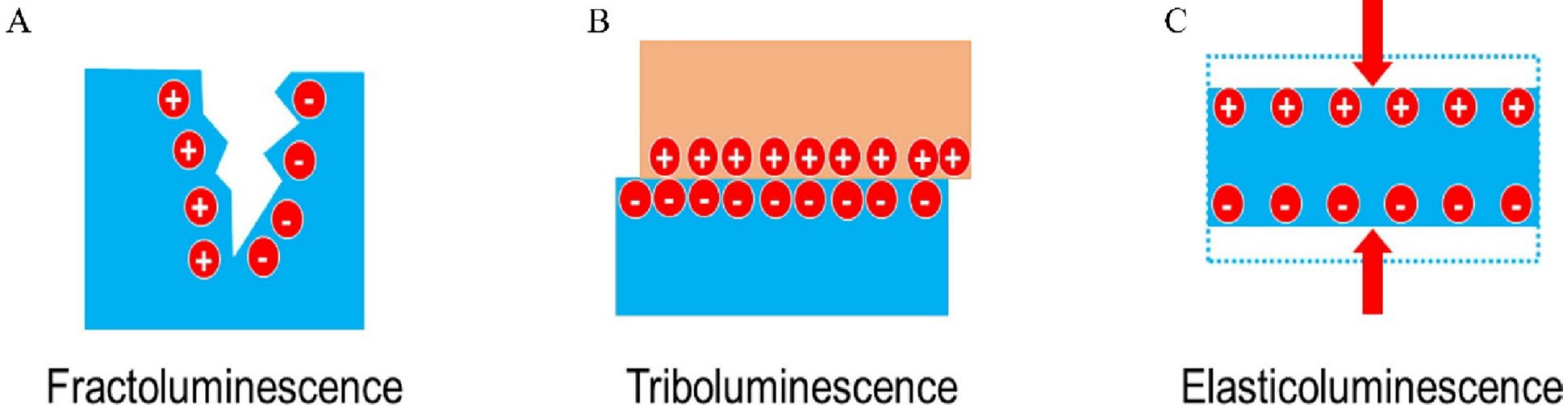
**A**) Fractoluminescence arises from the irreversible breaking of ionic and covalent bonds, emitting light through charge separation and recombination. **B**) Triboluminescence results from contact-induced charge transfer, often linked to friction or mechanical deformation **C**) Elasticoluminescence, a reversible process triggered by elastic strain, with emission intensity typically scaling linearly with applied stress, attributed to interactions between piezoelectric and electroluminescent effects. Reproduced with permission from [Yang *et al.*], “[Principles and applications of sono-optogenetics],” [Adv Drug Deliv Rev], © [2023], Elsevier

Certain crystalline materials emit photons (light) in response to mechanical stress [97]. Ultrasound can provide the required stress, either through pressure waves or via the vibration of a piezo-luminescent composite. Mechanoluminescent (ML) nanoparticles are designed to absorb mechanical energy and re-emit it as visible-range photons [98]​. If these photons match the activation spectrum of an optogenetic actuator (e.g. blue light for Channelrhodopsin-2), then ultrasound can indirectly trigger optogenetic stimulation – a technique termed sono-optogenetics​. Figure 4 summarizes three distinct mechanoluminescence phenomena—fractoluminescence, triboluminescence, and elasticoluminescence—highlighting their underlying mechanisms and relevance to stress-induced light emission in functional materials.

A landmark example is the work by Wu *et al.*, who developed ZnS: Ag, Co@ZnS core–shell nanoparticles that emit ~ 470 nm blue light under ultrasound excitation​. These ML nanotransducers were injected into the bloodstream of mice expressing Channelrhodopsin-2 (ChR2) in neurons. By shining a priming light (around 400 nm) on superficial blood vessels, the nanoparticles were “charged” optically (this step puts the crystal lattice into a metastable state). Subsequently, FUS was applied through the intact scalp and skull; wherever the ultrasound focus contained nanoparticles, they would light up in flashes of blue, stimulating nearby ChR2-expressing neurons​. This approach achieved wireless, deep brain optogenetic activation without any fiber optic implants – truly leveraging the synergy of nanotech and ultrasound. In the reported experiments, sono-optogenetic stimulation of the motor cortex in Thy1-ChR2 mice induced limb movements consistent with neuronal activation​. No tissue damage was observed from the nanoparticles or ultrasound in these conditions​ [99].

Several materials exhibit mechanoluminescence, including doped zinc sulfide (as above), strontium aluminates, or certain perovskites [100]. The efficiency (number of photons per mechanical input) and wavelength of emission are key metrics. The ZnS: Ag, Co system emits in the blue and was specifically matched to ChR2​, but by altering dopants one could tune emission to other wavelengths (potentially targeting red-shifted opsins like ChrimsonR or other light-sensitive proteins). The intensity of emission under FUS can be modest; thus, current research focuses on improving the brightness of ML nanoparticles and the coupling of their light to neurons. Strategies include optimizing the crystal doping levels [101–103], using core–shell structures to enhance light outcoupling [104], and using higher ultrasound pressures or resonance modes to stimulate a stronger luminescent response​. Another innovation could be to incorporate ML particles into hydrogels or scaffolds in the brain to keep them localized near target cells for longer-term use​ [105].

One challenge of sono-optogenetics is the need to “pre-charge” the ML particles with an excitation light (as in the above example). Some mechanoluminescent compounds require this step (they are called elastico-luminescent, needing a prior fill of trap states via UV/visible light). Others might emit upon stress without pre-illumination (crystalloluminescence), but their biocompatibility and brightness remain to be fully tested. Still, the concept has been proven: a circulating nanoparticle can act as an internal light source that is triggered by ultrasound on demand​ [99]. This effectively turns ultrasound into a means of 3D addressable light delivery. From a neuromodulation perspective, it combines the cell-type specificity of optogenetics (since only neurons with opsins will respond to the light) with the deep penetration of ultrasound.

#### Comparison with direct optical technique

In conventional optogenetics, light scattering in tissue limits the reachable depth (~ 1–2 mm for visible light) [106]. Sono-optogenetics circumvents this by using ultrasound as the delivery vehicle and generating light locally at the focus [107]. This opens possibilities for noninvasive activation of deep brain regions that were previously out of reach for optogenetics. It also allows multiplexing – potentially, different frequencies of ultrasound or different nanoparticle types could address different brain areas or circuits with distinct colors of light. The temporal resolution of sono-optogenetics is currently limited by the ultrasound pulse parameters (typically milliseconds to hundreds of milliseconds for safe pressures), which is slower than direct optical stimulation (which can be a fraction of a millisecond [108]). However, ongoing advances in transducer technology and feedback control may improve the temporal precision.

In summary, mechanoluminescent nanosystems add an optical modality to ultrasound neuromodulation. They exemplify the creativity of multimodal approaches: using acoustically triggered luminescence to achieve a biological effect (neuron activation via light-gated channels) that ultrasound alone could not directly induce. This modality is still in early stages, but it has demonstrated the principle of contact-free, implant-free deep optogenetics​ [99].

#### Applications of mechanoluminescent nanosystems for optogenetic neuromodulation

The concept of sono-optogenetics has been validated in animal models, and researchers are building on that foundation to improve and apply this technology. Wu *et al.* (2019) first demonstrated in mice that systemically delivered ZnS: Ag, Co@ZnS ML nanoparticles could enable FUS-driven optogenetic stimulation of the motor cortex​. In their experiments, mice expressing Channelrhodopsin-2 in motor neurons received an IV injection of the ML nanoparticles. Upon ultrasound sonication of the motor cortex (after an initial optical charging step), the mice exhibited stimulation of the contralateral limbs, consistent with motor cortex activation​. Electromyography and high-speed videography confirmed muscle contractions occurring in synchrony with the ultrasound pulses only when the nanoparticles and opsin were present​. This was effectively a wireless deep brain prosthesis without wires or optical fibers [99].

Since then, improvements have been made in the materials. For example, researchers have explored europium-doped materials that emit red light under stress, aiming to pair with red-shifted opsins (which penetrate further and cause less phototoxicity). There is also interest in nanoparticles that don’t require an external pre-charge light. A 2023 review by Yang *et al.* summarizes that new mechanoluminescent compounds (like perovskite nanocrystals in a polymer matrix) can have their trap states filled by ambient thermal energy or by the mechanical excitation itself, eliminating the need for UV charging​ [57]. While these are in early stage, they point to a future where a clinician could inject a formulation of ML nanocrystals, then simply apply a focused ultrasound transducer over the skull to activate a desired brain circuit with light all noninvasively.

A cascaded mechanoluminescent liposome-based nanotransducer has been developed by Wang and colleagues to achieve efficient light emission triggered by ultrasound, generating blue light with a subsecond response latency. By combining the high energy transfer efficiency of focused ultrasound in brain tissue with the exceptional sensitivity of these nanotransducers, efficient photon delivery was demonstrated, activating ChR2-expressing neurons in both the superficial motor cortex and the deep ventral tegmental area following intracranial injection [109].

An inverse approach was used by Li and coworkers who developed an optically-generated focused ultrasound (OFUS) system for non-invasive brain stimulation with ultrahigh spatial precision (~ 83 μm). OFUS is generated by a curved optoacoustic pad (SOAP) composed of candle soot nanoparticles embedded in PDMS, which converts nanosecond laser pulses into high-frequency ultrasound via the optoacoustic effect. This focused wave exerts mechanical radiation force at the neuronal membrane, likely activating mechanosensitive ion channels without thermal or cavitation effects. A single ultrasound cycle was sufficient to stimulate neurons both directly and transcranially in mice. Compared to conventional ultrasound neuromodulation, OFUS achieved similar neural activation with ~ 10,000× lower energy input. Functional responses were confirmed via calcium imaging, c-Fos expression, and EMG. The method offers a safe, scalable platform for precise neuromodulation and brain circuit mapping [110]. Similarly, A fiber optoacoustic converter (FOC) featuring a two-layer design to enable tunable frequency output was developed by Ying *et al.* The FOC was applied for photoacoustic neuromodulation, demonstrating efficacy in cultured rat cortical neurons as well as in the mouse brain. A 1030 nm laser with a 3 ns pulse width and 100 µJ pulse energy served as the excitation source, producing an acoustic frequency spectrum with peaks between 1 and 5 MHz. The 1/e decay length of the generated acoustic wave measured in water was approximately 1 mm, with temperature increases of 1.6 °C and 0.5 °C for 200 ms and 50 ms pulse trains, respectively. *In vivo*, local field potential (LFP) responses were observed for both 200 ms and 50 ms pulse trains, and LFP amplitudes decreased from 159.8 ± 13.2 µV to 10.5 ± 5.1 µV at a distance of 400 μm from the fiber, confirming spatial confinement. FOC stimulation also elicited motor responses consistent with prior studies [111].

Another application of ML nanoparticles is in mapping and imaging. When these particles emit light, it could be detected to infer where the ultrasound focus was or whether the nanoparticles have reached a target (a form of acoustic functional imaging). In one demonstration, particles emitting in the NIR (near-infrared) were used in a tissue phantom; ultrasound caused them to light up, and the light was detected by external sensors to verify targeting​ [99]. Though the brain is not transparent, fiber-based detectors or endoscopes could potentially capture emitted light from mechanoluminescent probes for real-time feedback. Table 6 provides an overview of recent studies utilizing mechanoluminescent nanosystems assisted with ultrasound for neuromodulation and therapeutic applications across various models.

**Table 6:** Summary of studies employing ultrasound-assisted mechanoluminescent nanosystems for neuromodulation and therapeutic applications

Reference	Nanosystem	Ultrasound parameters	*In vitro/in vivo*	Animal model/ cell type	*In vivo* target	Neuromodulatory effects	Therapeutic effects
Wang *et al.* (2023) [123]	Lipo@IR780/CaO_2_/L012 liposomes (chemiluminescent L012, sonosensitizer IR780, and sono-amplifier polyethylene glycol (PEG) 200 coated calcium peroxide (CaO_2_) nanoparticles loaded into lipids) D = 175.9 ± 0.6 nm Light emission = 470 nm	*f* = 1.5 MHz, *P =* 1.55 MPa, DC = 10% (100ms FUS on and 900ms FUS off)	Both	*In vitro:* Primary neurons transduced with AAV9-hSyn::ChR2- EYFP and AAV9-hSyn::NES-JRGECO1a. *In vivo*: Thy1-ChR2-YFP transgenic mice	Secondary motor cortex (M2) and Ventral tegmental area (VTA)	*In vitro*: •~ 80% synchronized firing in ChR2(+) neurons under FUS(+) and LipoCaO₂(+) conditions was observed. *In vivo:* • Contralateral limb motion with FUS + liposomes was observed while no ipsilateral limb motion was observed. • Elevated c-Fos expression in right M2 region. • Elevated c-Fos expression in right VTA region.	NA
Wang *et al. *(2023) [109]	Lipo@IR780/L012 (L012 and IR780 loaded into the lipids) D = 120.9 ± 0.5 nm	*f* = 1.5 MHz, *P =* 1.50 MPa, DC = 10% (100ms FUS on and 900ms FUS off)	Both	*In vitro:* CheRiff-eGFP tet-on spiking human embryonic kidney 293 (HEK) cells *In vivo:* Thy1-ChR2-YFP transgenic mice	Motor cortex region targeted following an intravenous injection of the NPs.	*In vitro:* • Increase in Ca2 + influx. *In vivo:* • c-Fos expression significantly increased in motor cortex. • Sharp changes in both hip to knee joint angle (θ) and in knee to feet joint angle (φ).	NA
Wu *et al.* (2019) [99]	ZnS: Ag, Co@ZnS coated with 1,2-distearoyl-sn-glycero-3-phosphoethanolamine-*N*-[methoxy(polyethylene glycol)-2000] (DSPE-mPEG) D = 86.6 ± 13.0 nm Light emission = 470 nm	*f* = 1.5 MHz, *P =* 1.18 MPa, DC = 10% (100ms FUS on and 900ms FUS off), PRF = 1 Hz, *I* = 10.0 W/cm^2^	Both	*In vitro:* NaV 1.3 KIR 2.1 HEK cells transfected with ChR2 *In vivo:* Male C57BL/6J mice and Thy1-ChR2-YFP mice	Secondary motor cortex (M2) was targeted following an intravenous injection of NPs.	*In vitro:* • Action potentials in ChR2(+) HEK cells under FUS via circulating mechanoluminescent nanoparticles were evoked. • Periodic single-unit spikes only in ChR2(+)/nanoparticle(+) group were detected. • Increased spike amplitude under repetitive FUS stimulation. *In vivo:* • Unilateral hindlimb motion in Thy1-ChR2-YFP mice was evoked via FUS targeting M2 cortex. • Contralateral limb motion synchronized with FUS pulses was detected; ipsilateral limb showed minimal movement.	NA

* *f* = Center frequency, PRF = Pulse repetition frequency, *P* = Acoustic Pressure, *t* = time duration, P_w_ = Power, D = Diameter, W = Width, L = Length, NP = Nanoparticles, US = Ultrasound, *I* = Intensity

#### Safety considerations

The ML nanoparticles used typically contain heavy metal dopants (Ag, Co, etc.) in a ZnS matrix. So far in small animal tests, no toxicity or organ damage has been seen from a single injection​ [99, 112]. However, long-term accumulation or repeated dosing needs evaluation. The particles might end up in the liver or spleen as many nanoparticles do. Future particles might use fully biodegradable systems that slowly dissolve after use (e.g. silica-based mechanoluminescent frameworks that release benign products). Another safety aspect is avoiding any significant heating.

#### Applications in neuroscience

From a neuroscientist’s perspective, sono-optogenetics offers a unique tool: one can stimulate deep brain regions in an intact animal, turn stimulation on/off rapidly, and do so without tethering the animal. This is incredibly useful for studying causality in brain circuits. Already, groups are using it to probe things like deep pain pathways or hypothalamic feeding circuits in freely moving animals, which were previously very hard to access with optogenetics. Moreover, because ultrasound can be scanned or steered electronically, one could envision scanning an ultrasound beam across the brain to “interrogate” different regions with light. Guo and colleagues called this “scanning optogenetics”​ [57]. This might allow high-throughput screening of brain functional connectivity by remotely activating one area after another in rapid succession.

In summary, mechanoluminescent nanosystems have proven that ultrasound can serve as a trigger for remote-controlled internal light sources. This combination of ultrasound and optogenetics leverages the strengths of each and mitigates the weaknesses (optogenetics’ limited penetration, ultrasound’s lack of cell specificity). While challenges remain in material optimization and ensuring a sufficient photon yield in the complex milieu of the brain, the path forward is clear. We anticipate that in the next few years, refined ML nanoparticles and perhaps new mechanophotonics approaches (like acoustic meta-materials that focus energy into emitters) can make sono-optogenetics even more practical. It stands as a prime example of FUS-assisted multimodal neuromodulation, fulfilling the vision of multidisciplinary neuroengineering that combines physics, nanochemistry, and gene therapy to achieve unprecedented control over brain activity.

### Ultrasound-triggered drug delivery for neuromodulation

#### Working principle

A different but complementary use of US and nanosystems is to achieve targeted neurochemical modulation. Instead of (or in addition to) directly stimulating neurons by physical means, ultrasound can be used to release or deliver neuroactive compounds at a desired site and time. This approach leverages decades of drug delivery research, repurposing it for neuromodulation. Two main classes of carriers have been explored: **(i)** phase-change nanodroplets/microbubbles that can release encapsulated drugs upon ultrasound-induced vaporization or oscillation, and **(ii)** nanoparticles that stably carry drugs and unload them when ultrasound triggers a structural disruption. There are several nanosystems that have been explored for this strategy.

##### Acoustic contrast agents as drug carriers

Acoustic contrast agents—including nanodroplets, nanobubbles, and microbubbles—are engineered to enhance ultrasound-mediated drug delivery by exploiting their phase-change and cavitation properties. These agents enable spatially precise payload release through mechanisms such as acoustic vaporization and acoustic cavitation, allowing for site-specific therapeutic interventions. Ultrasound parameters can be finely tuned to trigger drug release or mechanical bioeffects, optimizing neuromodulation and minimizing off-target exposure.

###### Nanodroplets

These are typically perfluorocarbon emulsions – liquid-core droplets at nanoscale (100–300 nm) that can be converted to gas microbubbles when hit by ultrasound. The liquid-to-gas transition (acoustic vaporization) causes a dramatic expansion, which can break the droplet shell and release any payload. Nanodroplets consist of two primary components: an outer encapsulating shell and an inner core containing liquid perfluorocarbon (PFC). The formulation of these components is a critical determinant of nanodroplet physicochemical properties. The shell is engineered to preserve the structural integrity and original diameter of the droplets following intravenous administration, while also permitting expansion into gas bubbles upon acoustic droplet vaporization (ADV). The effective surface tension of nanodroplets is largely governed by the physicochemical state of the shell. Low–boiling point perfluorocarbons can persist in a superheated liquid state at physiological temperatures as a result of the Laplace pressure exerted by the shell. This Laplace pressure arises from interfacial surface tension between the shell and the PFC core. Consequently, nanodroplets remain stable *in vivo* until sufficient acoustic energy is applied to induce vaporization via ADV [113].

###### Microbubbles and nanobubbles

While not delivering a neuromodulator per se, the use of microbubbles and nanobubbles with ultrasound to open the BBB is being tested to deliver therapeutic antibodies or gene therapies for neurological diseases (like anti-amyloid antibodies in Alzheimer’s). In principle, one could also open the BBB in a targeted region and systemically administer a neuroactive drug, allowing it to enter the brain only where needed. This indirect method might treat conditions such as epilepsy (by focally delivering an anticonvulsant) or depression (by delivering, say, a trophic factor) without systemic side effects. A recent clinical trial in Alzheimer’s disease showed that repeated FUS with microbubbles can safely open the BBB across large brain regions and even on its own led to reduced amyloid burden and improved neuropsychiatric symptoms​ [114]. This “FUS-BBB opening” strategy is somewhat adjunct to neuromodulation, but worth noting as part of the FUS toolbox. It exemplifies how ultrasound and microbubbles can noninvasively facilitate interventions (here pharmacological) in the brain that were previously only possible with invasive routes.

An advancement of the above concept is using pre-formed nanobubbles delivered to specific spots. In a Nature Communications 2024 study by Hou *et al.* (same group we discussed in the “Applications of Gas Vesicles for Neuromodulation” section), tiny gas nanobubbles were injected into precise brain regions in mice​. Ultrasound stimulation of these nanobubbles achieved sub-millimeter precision neuromodulation, as mentioned. While that study primarily focused on mechanical activation, it also demonstrated behavioral modulation relevant to neuropsychiatric states (depression-like behavior) ​ [95]. This suggests that by appropriate targeting (e.g. nanobubbles to the reward circuit or mood circuit), one can use ultrasound to induce beneficial circuit-level changes noninvasively – a form of sonodynamic therapy for brain disorders, where the “dynamic” agent is a bubble. Although no exogenous chemical was delivered in that case, the principle can be similar: nanobubbles could potentially be loaded with neuromodulators or gene vectors that release upon cavitation. The key advantage shown was the extreme spatial focus, overcoming the usual diffraction limit of ultrasound because the effect was limited to where bubbles were present.

##### Ultrasound-responsive drug-loaded nanoparticles

Beyond phase-change agents, solid or colloidal nanoparticles can be designed to release drugs under ultrasound via various mechanisms: acoustic cavitation can disrupt particle coatings, mild heating can melt matrices or liposomes, or mechanical vibrations can shake loose a drug tethered by a sonosensitive linker​​. These approaches strive for an “on/off” remote-controlled drug delivery with high spatial and temporal resolution. One example of such nanosystem is mesoporous silica nanoparticles (MSNs). These inorganic nanosystems exhibit a high drug-loading capacity owing to their intrinsic mesoporous structure, which significantly increases the available surface area. To prevent premature diffusion-driven release of the therapeutic payload, it is necessary to block the pores using molecules that function as pore-capping agents. These caps are designed to detach from the MSNs only upon exposure ultrasound (US)-induced mechanical stress thereby enabling controlled release of the encapsulated cargo [115]. Liposomes, nanoemulsions, polymeric micelles, and polymersomes can also encapsulate therapeutic cargo within their internal structures. In contrast to surface-loaded nanoparticles, their response to ultrasound is governed by the properties of the self-assembled system. For instance, temperature elevations induced by focused ultrasound (1.1 MHz) during hyperthermia treatments increase liposomal membrane permeability, thereby promoting payload release [116]. Ultrasound irradiation at lower frequencies and higher amplitudes produces shear stresses capable of rupturing vesicular structures. However, disrupted liposomes can rapidly reassemble into multiple smaller vesicles while preserving the total surface area, assuming no phospholipid loss. As a result, this process leads to only partial release of the encapsulated cargo [116, 117].

#### Applications of ultrasound-triggered drug delivery for neuromodulation

Several studies have employed the delivery of neuromodulatory agents as a neuromodulation strategy. A notable example is by Wilson *et al.* 2019, who formulated polymer-shelled nanoparticles containing a high-boiling-point perfluorocarbon (perfluorooctyl bromide, PFOB) core, loaded with the anesthetic drug propofol​​. The PFOB core made the particles stable in circulation (they would not gasify spontaneously) but responsive at higher acoustic pressures. In macaque monkeys, they injected these propofol-loaded nanoparticles IV and applied focused ultrasound to the lateral geniculate nucleus (LGN) in the thalamus – a relay for visual information. During one-minute sonications, over 80% of the propofol payload could be released when pressures exceeded 1.3 MPa. The functional effect was striking: the monkeys were doing a visual choice task, and after ultrasound-mediated propofol release in the right LGN, they showed a strong bias toward choosing the right-side target (meaning the right LGN – responsible for left visual field – was inhibited by propofol, so they missed left targets)​​. This reversible functional lesion confirmed that the drug was indeed delivered and active at that deep brain site, modulating the animal’s behavior​. Because ultrasound targeting could be alternated between left and right LGN, they were able to modulate visual perception in a lateralized manner on demand​. Crucially, control experiments showed that ultrasound alone (with empty particles) did not cause such biases​, and propofol systemically without FUS would normally cause global sedation (which was not observed). Thus, the effect was due to localized drug uncaging. This study, conducted in a large-brain animal, is a strong proof-of-concept that FUS-triggered drug delivery can perform targeted neuromodulation in sophisticated behavioral paradigms​ [118]. It paves the way for eventually using this approach in humans – for example, delivering an anti-epileptic drug to a seizure focus at seizure onset, or releasing a neuromodulator in a specific nucleus to treat pain or mood disorders.

In another study, Wang *et al.* (2021) used polymeric nanoparticles (PLGA-PEG) loaded with propofol and demonstrated ultrasound-triggered release at a threshold of about 0.8 MPa using a 650 kHz US transducer *in vitro*. They applied these to a rat brain slice preparation and showed that ultrasound bursts could suppress evoked neural activity (visual evoked potentials) when the propofol was released, with the suppression reversing quickly after sonication stopped​​. This rapid on/off effect is desirable for a neuromodulation tool. It means one could, in principle, modulate neural circuits for a short duration and then allow them to resume normal function, as opposed to a systemic drug which affects the whole brain for hours [119]. Similarly, Wang and colleagues developed a novel class of porous hydrogen-bonded organic frameworks (HOFs) that can be precisely triggered by focused ultrasound (FUS) to release therapeutic cargo in deep tissues. Using theoretical modelling they correlated the ultrasound peak pressure with the dissociation kinetics of the HOFs and demonstrated ultrasound-programmable drug release thresholds (from ~ 0.5–8 MPa depending on HOF type). *In vivo*, the HOFs loaded with the designer drug clozapine-N-oxide (CNO) were delivered into the ventral tegmental area (VTA) of rodents, where FUS (1.5 MHz, ~ 1.4–2.5 MPa) induced rapid (in seconds) release of CNO, activation of hM3D(Gq)-expressing neurons (via GCaMP monitoring) and modulation of behaviour. The system was shown to be biocompatible, with minimal thermal effects (rise < 1.3 °C) and no overt tissue damage. Overall, this work demonstrates a mechanoresponsive material platform enabling non-invasive, spatio-temporal control of molecular therapeutics in deep brain regions using ultrasound [120]. Furthermore, nanodroplets as carriers for neuromodulatory drugs have also been employed *in vivo.* Lea-Banks *et al.* developed < 220 nm lipid-coated decafluorobutane nanodroplets loaded with a sedative drug (pentobarbital) to achieve either stimulation or suppression of brain activity with FUS​​. In rat experiments, they sonicated the motor cortex with 1.66 MHz FUS, using a feedback control to trigger bursts when cavitation (vaporization) was detected​. Sham nanodroplets (no drug) caused a localized stimulating effect. This was likely due to the mechanical action of bubble formation, evidenced by a 22% increase in c-Fos expression in the sonicated area. In contrast, pentobarbital-loaded nanodroplets caused a 22% decrease in c-Fos, indicating neuronal suppression/anesthesia from the released drug​​. Importantly, neither case caused BBB disruption or damage, thanks to the feedback control that stopped ultrasound once vaporization occurred. This study illustrates a versatile “tuneable” neuromodulation by simply changing whether the nanodroplet carries an excitatory drug, inhibitory drug, or no drug, one can achieve different neuromodulatory outcomes with the same ultrasound setup​ [121]. It also underscores the ability of FUS to localize drug action – the effects (measured by c-Fos and behavioral tests) were confined to the targeted cortical area.

#### Potential and challenges

Ultrasound-responsive drug delivery combines chemistry with physics: one can choose virtually any neuromodulatory compound – neurotransmitters, receptor agonists/antagonists, gene editors, etc. – encapsulate it and then use ultrasound as the trigger to release it at the target. It offers chemogenetic-like specificity (using specific drugs) but with spatiotemporal control (thanks to FUS). For instance, one could release a GABA agonist to inhibit a region, or a glutamate receptor blocker to selectively block a pathway, or even trophic factors to promote plasticity in a targeted manner. A recent insight is that such localized pharmacological modulation can also have network-level effects: by inhibiting one node (say LGN), one can observe how connected regions’ activity changes, thus mapping functional connectivity​. This suggests a use in basic neuroscience to study brain circuits by “remote-controlled pharmacology.”

However, there are challenges: ensuring the nanoparticles actually reach the brain region of interest (systemic IV injection relies on some accumulation or BBB crossing). Many studies bypassed this by direct injection of the particles into brain tissue​. To move to noninvasive delivery, one might combine FUS-induced BBB opening with nanoparticle delivery – open the BBB in a target area, let the particles extravasate there, then on a second ultrasound pass, trigger the release. This two-step FUS might be complex but is within current technical capability. Another challenge is controlling dosage: one needs to calibrate how much drug is released with a given ultrasound exposure to avoid too much (or too little) drug. Feedback mechanisms (e.g. analyzing acoustic emissions to gauge cavitation) might help deliver consistent doses​ [122].

Moreover, the pharmacokinetics in the brain after release need study – e.g. propofol released in LGN diffused and was presumably taken up or metabolized locally without significant global effects​ [118], but this may vary with drugs. Encouragingly, in the primate study, the neuromodulatory effect was transient and tied to the presence of ultrasound; behavior normalized minutes after sonication, implying the drug didn’t linger at high concentration systemically​.

In summary, ultrasound-activated drug delivery is a potent form of multimodal neuromodulation that taps into the rich pharmacology of the brain. It can achieve effects that are impossible with physical stimuli alone (since drugs can mimic or block endogenous neurotransmitters with high specificity). By combining this with spatial precision of FUS, one essentially has “remote-controlled drug injections” in the brain. The latest breakthroughs in noninvasive BBB opening and nanoparticle engineering (such as stimuli-sensitive liposomes and hydrogels) bolster the feasibility of this approach. It holds promise not only for research (e.g. reversible inactivation of a small brain region to test its function) but also for therapy (e.g. targeted delivery of antiepileptic drugs, dopamine for Parkinson’s, or local anesthetics for pain). Table 7 provides an overview of recent studies utilizing FUS-triggered drug delivery in neuromodulation contexts, illustrating the range from rodents to non-human primates.

**Table 7:** Summary of studies employing ultrasound-assisted nanosystems for the delivery of neuromodulatory drugs

Reference	Nanosystem	Ultrasound parameters	*In vitro/in vivo*	Animal model/ cell type	*In vivo* target	Neuromodulatory effects	Therapeutic effects
Wang *et al.* (2025) [120]	Hydrogen-bonded organic frameworks (HOFs) encapsulated with clozapine N-oxide D = 250 to 600 nm	*In vitro:* *f =* 1.5 MHz, *P =* 1.08 MPa, *t =* 10s *In vivo:* *f =* 1.5 MHz, *P =* 1.40 MPa, *t =* 20s	Both	*In vitro:* Primary cortical neurons *In vivo:* C57BL/6 mice and Long–Evans rats	Intracranial Injection in the VTA	*In vitro:* • >90% of hM3D(Gq)+ neurons were activated following ultrasound stimulation, with a latency of ~ 1.6 s and sustained depolarization for ~ 60s. *In vivo:* • *In vivo* fibre photometry in mouse VTA showed increase in Ca²⁺ signal. • High temporal precision, with ~ 3.5 s activation latency was observed.	Treated rats exhibited a preference to stay in the conditioned chamber compared with other groups in post-tests.
Wilson *et al.* (2023) [118]	Fluorocarbon-perfluorooctyl bromide (PFOB) filled nanocarriers loaded with propofol D = 526 ± 109 nm	*f* = 480 kHz, *P* = 1.2–1.5 MPa, pulse length = 10–100 ms, DC = 10%, PRF = 3.33–10 Hz, *t* = 1 min	*In vivo*	Male rhesus macaques	Left and Right lateral geniculate nuclei (LGN)	• Ipsilateral bias in visual choices was induced following FUS-triggered drug release in LGN. • Significant side-specific neuroinhibitory effects confirmed at both 1.2 MPa and 1.5 MPa pressures. • Behavioral shift polarity matched expected effects of propofol.	NA
Banks *et al.* (2021) [121]	Definity based Nanodroplets with lipid shell (DPPC: DPPA: MPEG5000 DPPE) and liquid DFB core, loaded with pentobarbital (25 µg/mL) D = 212.5 ± 2.0 nm	*f* = 1.66 MHz, *P* = 1.1 MPa, pulse length = 10 ms, PRF = 1 Hz, *t =* 180s	*In vivo*	Male Sprague Dawley rats	Motor Cortex	• 21.7 ± 13% reduction in c-Fos positive cells. • Gait changes observed 90 min after FUS indicating asymmetric anesthesia of the motor cortex. • Stumbling on the ipsilateral side, overlapping paw prints on the affected side, increased paw angle and faint paw prints on the contralateral side.	NA
Banks *et al.* (2020) ([122]	Pentobarbital-loaded nanodroplets D = 210 ± 80 nm	*f* = 580 kHz, *P* = 0.32 MPa (increased by 6.4 kPa each second until drop vaporization was detected), pulse length = 10 ms, PRF = 1 Hz, *t =* 180s	*In vivo*	Male Sprague Dawley rats	Motor Cortex	19.1 ± 13% motor deficit on the contralateral side.	Successful local anesthesia.
Wang *et al. *(2018) [119]	PEG-PLGA micelles loaded with propofol D = 397.3 ± 10.0 nm	*f* = 650 kHz, *P* = 0.5–1.8 MPa, pulse length = 100 ms (60 times), PRF = 1 Hz,	*In vivo*	Long-Evans Wild-Type rats	Intravenous injection in the tail vein followed by ultrasound sonication in primary visual cortex (V1), motor cortex (M1) and LGN	• Reduction in EEG power (total and theta bands). • VEP N1P1 amplitudes significantly reduced. • M1 showed no attenuation of N1P1 VEP (no nonspecific effect). • Significant attenuation the VEP amplitude in LGN (smaller secondary effects). • FDG-PET imaging showed decreased glucose uptake. • Secondary regions of decreased FDG avidity outside the primary sonication site.	NA
Airan *et al.* (2017) [178]	Propofol loaded Nanoemulsions with liquid perfluoropentane core D = 320 ± 150 nm	*f* = 1 MHz, *P* = 1 MPa followed by 1.5 MPa after a 10 min interval, pulse length = 50ms, PRF = 1 Hz, *t* = 60s	*In vivo*	Adult male Fischer 344 rats	Intravenous Injection in the tail vein followed by ultrasound sonication focus was developed ∼5 mm caudal to bregma	Significant declines of total and theta band EEG power.	NA

*f = Center frequency, PRF = Pulse repetition frequency, *P* = Acoustic Pressure, *t* = time duration, P_w_ = Power, D = Diameter, W = Width, L = Length, NP = Nanoparticles, US = Ultrasound, *I* = Intensity, DPPC = Dipalmitoylphosphatidylcholine, DPPA = 1,2-palmitoyl-phosphatidic acid, MPEG5000 DPPE = R)-∝-[6-hydroxy-6-oxido-9-[(1-oxohexadecyl)oxy]-5,7,11-trioxa-2aza-6-phosphahexacos-1-yl]- ω-methoxypoly(ox-1,2-ethanediyl), DFB= Decafluorobutane, PLGA = Poly(lactic-co-glycolic acid)

## Conclusion and outlook

Ultrasound combined with specialized nanosystems has unlocked new possibilities for precise, multimodal neuromodulation that were unattainable with conventional techniques. Compared to electrical, magnetic, or optical methods, ultrasound offers noninvasive access to deep brain regions with millimeter precision​. By itself, ultrasound can modulate neural activity, but the advent of acoustic-responsive nanoparticles amplifies and diversifies its neuromodulatory capabilities – enabling electrical, mechanical, optical, and chemical interventions all via a single external energy source. This convergence of technologies – ultrasound physics, nanomaterials, neurobiology – exemplifies the interdisciplinary innovation needed to address challenging neurological disorders.

### Summary of progress

Over the past decade, we have seen laboratory demonstrations of US-nano neuromodulation progress from *in vitro* experiments to *in vivo* successes in animal models. Piezoelectric nanoparticles have evoked action potentials in neurons and even improved functional recovery in nerve injury models via activity-dependent mechanisms. Gas vesicles and nanodroplets have achieved neuron activation in living brain with unprecedented spatial targeting, down to sub-mm regions and specific behaviors modulated​ [95]. Mechanoluminescent particles have established a new modality of sono-optogenetics, removing the depth barrier of optogenetics and allowing wireless control of genetically defined cells​ [123]​. Ultrasound-triggered drug release has been validated in non-human primates to produce reversible, focal neural inhibition without systemic effects ​ [118]. These achievements underscore the potential impact of US-assisted multimodal neuromodulation: therapies that are minimally invasive yet highly targeted and adaptable.

### Challenges and considerations

**Fig. 5:**
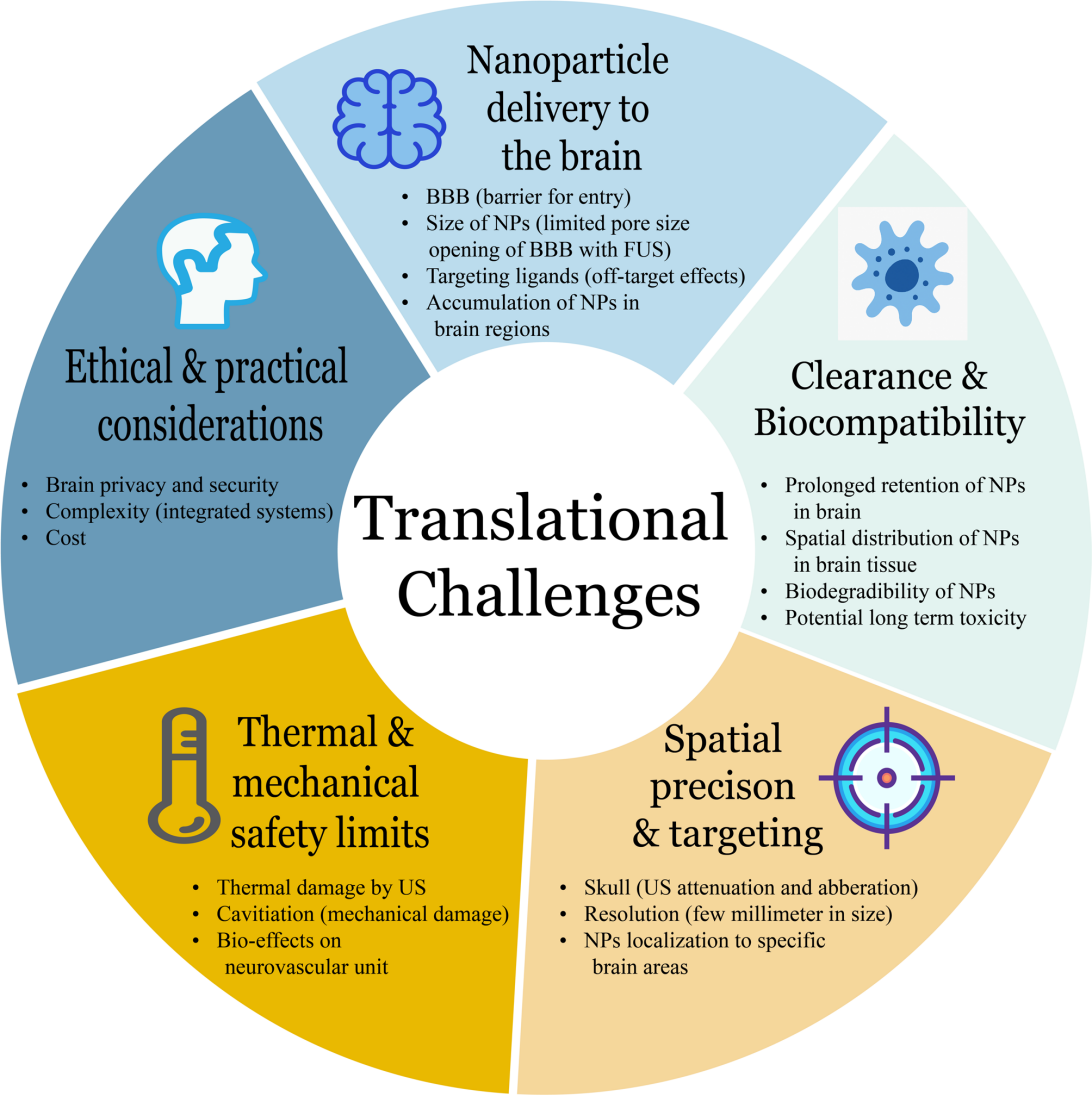
Schematic representation of the current challenges faced in the field of FUS assisted neuromodulation using nanosystems

Despite encouraging results, several challenges remain before clinical translation. We discuss some of the challenges in this review. A summarized scheme of the challenges is also presented in Fig. 5.

#### Nanoparticle delivery to the brain

Systemic intravenous delivery of nanoparticles faces the obstacle of the BBB. FUS-induced BBB opening (with microbubbles) can transiently increase permeability, but particle size is critical – typically, pores opened are on the order of tens of nanometers [124]. Thus, large nanoparticles (> 100 nm) might still not enter easily. Most studies to date used local (intracranial) injections of nanoparticles to ensure they reach the target. While these injections are less invasive than chronic implants and can be done through a small burr hole or via intraventricular routes, they are still invasive. To achieve effective neuronal targeting, the design of nanoparticles plays a critical role, particularly regarding the selection of ligands, as well as the size and shape of the nanoparticles. Typically, nanoparticle size especially those ranging from 10 to 100 nm are typically most effective for crossing the blood–brain barrier (BBB) and achieving neuronal uptake. Particles at the lower end of this range (10–20 nm) are particularly efficient at BBB penetration, as they can utilize transcytosis pathways while avoiding rapid clearance via renal filtration. This can allow us to deliver US responsive nanoparticles non-invasively [125]. Small piezoelectric nanoparticles can be utilized to overcome this challenge. Receptor mediated transcytosis is widely employed to deliver nanoparticles to the brain. Peptide ligands, antibodies, aptamers, and small molecules provide distinct advantages depending on the target receptor and the required specificity. Nanosystems such as drug-loaded nanoparticles, nanobubbles and microbubbles are widely decorated with targeting ligands to aid the nanoparticle delivery and to increase the specificity of nanoparticles to the neurons [126]. To reduce off-target effect induced safety concerns, the target receptor should be highly expressed in the endothelial cells of the brain vasculature, while being minimally expressed in other vascular endothelial cells. However, to date, nearly no receptor can meet this criterion [127]. Similarly, transferrin receptor mediated transcytosis (Tfr) is highly debated. Early studies using cultured cells and *in situ* brain perfusion models revealed that nearly 90% of transferrin was recycled back to the luminal side after endocytosis by brain endothelial cells, while only about 10% of transferrin was transported into the brain [128]. Recent studies have demonstrated that factors such as the affinity and valency of the TfR antibody, along with the ligand density on the nanoparticle surface, play crucial roles in regulating intracellular TfR trafficking and nanoparticle transport [129–131].

Intranasal drug delivery offers an efficient, noninvasive strategy to target the brain, circumventing many limitations associated with parenteral administration. Through intranasal administration, drugs can reach the brain via the olfactory mucosa, either by diffusing through the connective tissue surrounding the olfactory nerve bundles or along the olfactory nerve axons, thereby bypassing the BBB and could be potentially used in the delivery of neuromodulatory nanosystems to the brain [132].

Future research should focus on optimizing particle size, surface chemistry (e.g. coating with targeting ligands or cell-penetrating peptides), and use of sequential FUS BBB opening to achieve sufficient nanoparticle accumulation in the target region without surgical injections. One promising direction is developing ultrasmall yet efficient piezoelectric or mechanoluminescent particles (e.g. <50 nm) that could pass through BBB openings. Another approach is cell-based delivery: loading nanoparticles into circulating cells (like neutrophils or macrophages) that naturally migrate to injury or inflammation sites in the brain, then using FUS at those sites. A detailed review has been provided by Liu and coworkers discussing the strategies and challenges of nanoparticle delivery to the brain [127].

#### Clearance and biocompatibility

Once nanoparticles have done their job, what becomes of them? The brain does not readily eliminate solid particles; they can persist for long periods [133]. Activated microglia (the brain’s immune cells) may engulf nanoparticles, as observed in some studies​. In peripheral organs, nanoparticles are often cleared by macrophages in the liver/spleen, but in the brain, they might stay *in situ* or drain slowly via glymphatic pathways [134]. Gu *et al*. studied the clearance of two different nanosystems, high-density lipoprotein (rHDL) nanoparticles (22.69 ± 1.97 nm) and poly(ethylene glycol)-b-poly(lactic acid) (PEG-PLA) (94.11 ± 3.52 nm) nanoparticles. The authors demonstrated that both types were rapidly eliminated from the mouse brain, exhibiting a half-life of less than five hours. Quantitative analyses indicated that approximately 80% of total nanoparticle clearance occurred through the glymphatic route, emphasizing this system’s dominant role in eliminating exogenous nanoscale materials. Mechanistic investigations further revealed that microglia facilitated nanoparticle transport toward perivascular spaces, thereby enhancing clearance efficiency. Notably, when the glymphatic system was impaired, as modeled in Alzheimer’s disease mice, the rate of nanoparticle elimination was significantly reduced. These findings collectively establish the glymphatic network as a major pathway for nanoparticle drainage from the central nervous system and highlight the importance of considering clearance mechanisms in the design of brain targeted nanomedicines. This study suggests that the physicochemical properties of nanoparticles (size, surface chemistry, and degradability) should be carefully optimized not only to enhance brain delivery and targeting, but also to ensure their efficient and safe clearance from brain tissue, especially under pathological conditions where glymphatic function is impaired [134]. Another cited study found that when antibody labelled nanoparticles were injected into a tumor, most were later found in macrophages rather than tumor cells [135]​. Although that was a tumor context, it highlights the need to examine where the particles go over time and whether they induce inflammation. Biodistribution studies for each nanoparticle type will be important: e.g. are piezoelectric nanoparticles broken down or do they form aggregates? Do gas vesicle remnants get metabolized or excreted? Fortunately, many of the materials used (like lipids, proteins, polycaprolactone and PLGA polymers) are biodegradable. Self-assembling peptide nanofibers represent a novel and promising approach in neurology. These nanofibers could be functionalized with signaling molecules that can positively regulate neural stem cell migration, differentiation, and axonal growth [136]. Inorganic materials (BaTiO₃, ZnS) are not biodegradable, but they could potentially dissolve extremely slowly. For instance, barium titanate might leach barium ions over years. We need to ensure long-term safety, especially if multiple doses or chronic use is envisioned. Development of biodegradable or excretable nanotransducers is a high priority. For example, one could use bioresorbable piezoelectric polymers or silicon-based nanoparticles that dissolve. Another strategy is to make the nanoparticles sufficiently small so that after fulfilling their purpose, they can exit the brain to bloodstream during subsequent BBB openings or via CSF clearance.

#### Spatial precision and targeting in humans

The human brain is larger and the skull thicker than in rodents. Achieving focused ultrasound targets in humans requires large aperture transducers, phased arrays, and advanced beamforming (often guided by MR imaging). The skull is highly attenuating and aberrating. In addition, the pores located in the trabecular bone act as scatterers for ultrasound. As a result, ultrasound beams are both spatially shifted and attenuated. The spatial precision depends on frequency and aberration correction of the skull. It is essential to perform transcranial simulations which can predict the acoustic dose, the focalization characteristics, and provide insights for operation planning [137]. When combined with a multielement transducer (as seen in Fig. 2) or an acoustic lens [138], simulations can be used to correct the phase and amplitude (especially at high frequencies where the distortion induced by the skull is higher) of the emitted signal in order to achieve higher spatial precision through the skull [139, 140]. The spatial precision will depend on frequency and aberration correction of the skull. Current clinical low-intensity FUS systems can target regions a few millimeters in size, but not much smaller. The use of nanostructures, however, enhances the effective precision as seen with nanobubbles (since only where nanoparticles reside do we see an effect, even if some ultrasound energy spills around)​. For clinical translation, methods to localize nanoparticles to the intended target will be needed. This could be through convection-enhanced delivery (infusing them through a catheter to coat a region) or future molecular targeting (e.g. nanoparticles that bind to a particular cell type or lesion type). There is also interest in acoustic holography or meta-material lenses to create multiple simultaneous foci or custom-shaped focal volumes; combined with spatially distributed nanoparticles, this could modulate complex brain networks in synchrony. But such sophistication has yet to be realized in practice. In near-term, small discrete targets (like an epileptic focus, or a deep nucleus) are the likely candidates.

#### Thermal and mechanical safety limits

There are two main biophysical risks associated with the application of ultrasound: mechanical and thermal bioeffects [141]. Mechanical bioeffects mainly concern the risk of acoustic cavitation, which can lead to local tissue damage such as cell death or blood vessel hemorrhage. Thermal bioeffects may occur when mechanical energy is converted into thermal energy through absorption, leading to tissue heating and potential thermal damage [142]. While low-power US is generally safe with no tissue heating or damage if within recommended I_SPTA_/I_SPPA_ limits (as mentioned in the “Fundamentals of Ultrasound” section), the introduction of nanoparticles can, in some cases, increase risk. For instance, inertial cavitation of microbubbles can damage vessels or tissue if uncontrolled. If a nanoparticle strategy relies on strong cavitation (for drug release or BBB opening), careful real-time monitoring (via passive acoustic detection) and feedback control must be in place to stay within safe bounds [143, 144]. So far, neuromodulation studies have not reported adverse effects, but scaling up intensity or pulse duration could change that.

Ultrasound-induced temperature rise is dependent on several factors, including tissue properties (e.g., absorption of acoustic energy, tissue density and blood perfusion) [145], ultrasound exposure parameters (e.g., frequency, pressure amplitude, pulse duration, intensity and pulse repetition frequency) [146], and beam and scanning configurations [147]. Due to the high acoustic impedance of the skull, thermal considerations must be given not only to heat deposition at the intended deep brain target but also to cortical regions near the skull surface. Although skin, muscle, fat, and bone are generally more resistant to thermal damage than brain tissue, heating of the skull and scalp remains a critical safety concern [148]. In transcranial applications, the majority of heat is expected to be deposited at the outer surface of the skull [149, 150]. Moreover, heat accumulated within the skull can diffuse into adjacent tissues, including the brain, and continue to radiate and transfer thermal energy even after the end of the ultrasound pulse. Recent simulations based on non-human primate data illustrated the differential heating of skull and brain tissue, with temperature increase of 0.5 °C at the target site and 2.9 °C at the skull surface beneath the transducer, using a 250 kHz transducer, I_spta_ of 7.2 W/cm^2^, a 30% DC and a PRF of 10 Hz [151]. Application of FUS can also cause erythema, swelling, or burns on the skin. However, a recent study found no such clinical findings in human subjects with I_SPTA_ ranging from 6 W/cm^2^ to 12 W/cm^2^ (above the FDA guidelines of I_SPTA_ = 720 mW/cm^2^) [152]. Furthermore, the influence of FUS on the cerebrospinal fluid (CSF) needs to be taken into consideration. Slominski and colleagues studied the influence of CSF on power absorption during transcranial magnetic resonance-guided FUS treatment. Using Hybrid Angular Spectrum (HAS) simulations, treatment planning phase determination using morphologically realistic CSF and brain anatomy they reported an increase of up to 29% in the ultrasound focal absorbed power density using a 650 kHz transducer and a total power of 1 W [153].

Thermal changes during focused ultrasound treatments can be monitored using invasive and non‑invasive approaches. Invasive methods include thermal probes such as thermocouples and fiber-optic sensors that directly measure local temperature within tissue [154–156]. Non‑invasive methods primarily rely on imaging-based techniques, including magnetic resonance thermometry using proton resonance frequency shift [157], ultrasound-based thermometry, which estimates thermal changes via alterations in tissue acoustic properties [158] and computed tomography (CT) thermometry [159]. These approaches primarily assess tissue response rather than temperature itself. For example, contrast-enhanced imaging is used to visualize non-perfused (ablated) tissue, magnetic resonance and ultrasound elastography detect thermally induced changes in mechanical properties, and optical probes measure alterations in tissue optical characteristics. Collectively, these techniques provide real-time information on temperature distribution, thermal dose, and tissue effects, thereby supporting safe and effective treatment [154].

The International Transcranial Ultrasonic Stimulation Safety and Standards consortium (ITRUSST) has established consensus on considerations for nonsignificant biophysical risks. For mechanical effects, it is non-significant risk if the mechanical index (MI) or the mechanical index for transcranial application (MItc) does not exceed 1.9. For thermal effects, it is non-significant risk if any of the following three levels are met: the peak temperature rise does not exceed 2 °C or the peak absolute temperature does not exceed 39 °C, assuming a baseline temperature of 37 °C, the thermal dose does not exceed 2 CEM43 in brain tissue, 16 CEM43 in bone tissue, and 21 CEM43 in skin tissue, or specific values of the thermal index (TI) for a given exposure time [142].

Regulatory approval will require demonstrating that the combined ultrasound+nanoparticle treatment does not induce hemorrhage, unintended BBB disruption, or neurotoxicity. This might involve extensive animal testing and dose-escalation studies. It is encouraging that a recent human trial of FUS BBB opening in Alzheimer’s (using Definity microbubbles) across multiple sessions showed no serious adverse events​ [114], indicating the safety of repeated ultrasound + microbubble exposure, at least at the levels used.

Nevertheless, low intensity FUS can be strategically used to elevate tissue temperatures to induce thermal neurostimulation. A sonothermogenetic approach has been used by Yang and colleagues where they used low-intensity FUS to generate a pulsed wave with a 40% DC, a peak negative pressure of 1.3 MPa and an SD of 15s that elevates the tissue temperature up to 42 °C. The temperature rising activates neurons that have been genetically selected to express the thermosensitive ion channel TRPV1. This strategy enables noninvasive, cell-type-specific, and temporally precise modulation of neural activity in rodent models, while maintaining a favorable safety profile [33].

Furthermore, focused ultrasound combined with microbubbles (FUS + MB) can also influence brain cells beyond the vascular endothelium. Accumulating evidence demonstrates that the effects of FUS + MB extend to all major cellular components of the neurovascular unit (NVU), which comprises endothelial cells, pericytes, astrocytes, microglia, and neurons. This broad cellular involvement gives rise to a range of systemic bioeffects, including the induction of sterile inflammation, enhancement of metabolic and waste clearance pathways, and even stimulation of neurogenesis. Despite these widespread influences on brain physiology, a comprehensive understanding of the precise biological responses elicited in each NVU cell type remains incomplete [160].

#### Ethical and practical considerations

The idea of remote-controlled neural modulation raises exciting possibilities (e.g. neural prosthetics without wires), but also ethical questions about brain privacy and security if such technology became external and programmable. These are far on the horizon, but worth pondering early. Practically, delivering these therapies in a clinical setting might involve an integrated system: an MRI or ultrasound neuronavigation to position the beam, and perhaps an injection or IV infusion of the nano-agent, followed by monitoring. The complexity and cost need to be justified by significant clinical benefit. Thus, initial applications might be in severe, intractable conditions (e.g. refractory epilepsy, severe depression or OCD, chronic neuropathic pain, etc.) where current treatments fail or require highly invasive procedures. Demonstrating that US-nano approaches can achieve results on par with, say, DBS but without surgery would be a game-changer.

### Future Directions

There are several exciting avenues to explore:

- **Closed-Loop Neuromodulation**: Incorporating real-time feedback (e.g. EEG, functional ultrasound imaging, or magnetic sensors) to adjust US parameters. Nanoparticles could even be engineered as sensors (e.g. magnetothermal nanoparticles that report local neural activity) to create a closed loop where the brain’s response to stimulation is monitored and the next stimulus adjusted accordingly.
- **Multiplexed Control**: Using multiple types of nanoparticles that each respond to different ultrasound frequencies or pulse codes, allowing independent modulation of multiple regions or pathways simultaneously. Early studies have shown frequency-specific responses (e.g. nanodroplets vaporizing at certain frequencies) [161]; combining these could lead to an “ultrasound addressable neural network” concept.
- **Gene Therapy Synergy**: US can also facilitate gene delivery across the BBB or to specific cells (sonoporation). One could use FUS to deliver genes that make neurons more susceptible to mechanical stimuli (like overexpressing Piezo1 channels – a concept termed “sonogenetics”) [162]. This would reduce the needed ultrasound intensity and increase specificity. Already, *C. elegans* neurons engineered to express mechanosensitive channels have been stimulated by ultrasound in a dish​ [163]. A mammalian equivalent might use viral vectors plus US to target their uptake.
- **Crossmodal Synergy**: Combining US assisted neuromodulation with nanosystems with other neuromodulatory stimuli such as electrical, magnetic and chemical could open new avenues in this field. In the present, researchers have used a crossmodal approach by utilizing localized forces for nongenetic neuromodulation via light using either photoacoustic mechanisms or photoresponsive molecular actuators. In photoacoustics, millikelvin-scale temperature increase trigger ultrasound emission through thermoelastic expansion. When a laser is used to heat a material, if the optical pulses are shorter than the time scale of both thermal diffusion and sound propagation in the tissue, then a pressure wave can be generated [164].
- **Clinical Trials**: We anticipate that within the next 2–3 years, the first clinical trials of US neuromodulation with a chemical assist (e.g. FUS-facilitated drug to a focus) might occur. For instance, a trial might test FUS-triggered release of an anti-epileptic in patients with focal epilepsy, evaluating if seizures can be halted on demand. Other early trials might combine FUS with microbubbles in psychiatric indications, as some are already ongoing for depression (low-intensity FUS to frontal cortex) ​ [11]. While FUS itself has already entered clinical trials and its safety standards are under investigation [149, 150, 165], the addition of nanoparticles must be thoroughly validated. Specifically, each individual nanosystem must be independently assessed for safety and therapeutic efficacy in the context of ultrasound-mediated neuromodulation. Although certain polymeric nanosystems have already advanced to non-human primate models [118], most nanoparticle platforms remain at earlier stages of preclinical development and have been tested only in murine models. The translation of these nanosystems for ultrasound-mediated neuromodulation into clinical trials relies on the demonstration of biocompatibility, safety, clearance and therapeutic potential of the nanoparticles in combination with US. Before clinical application, it is essential to comprehensively characterize how these nanoparticles interact with biological systems, including potential toxicity, immune responses, and long-term accumulation *in vivo*. Equally important is the quantitative *in vivo* characterization of neuromodulatory effects at the cellular level, which is highly specific to each nanosystem. For instance, in the case of piezoelectric nanoparticles, it is essential to quantify the electrical stimulation generated per particle and to elucidate how this stimulation translates into neuronal activation, synaptic modulation, and network-level effects [166]. Such biological and mechanistic understandings are crucial to predict both efficacy and potential adverse effects in clinical applications. In addition, prior to any human applications, dedicated platforms specific to each FUS–nanosystem combination must be further developed and validated in animal models to enable safe and controlled treatment planning. Therefore, although the path ahead remains long, preclinical studies and in silico investigations will pave the way for the future integration of these technologies into clinical applications.

In conclusion, ultrasound combined with nanosystems represents a frontier of precision neuromodulation. It unites advances across domains – from the physics of wave propagation to the molecular design of nanocarriers – in service of modulating the brain in a controlled yet noninvasive manner. While challenges remain, the progress to date has been remarkable and continues to accelerate. The vision of remotely tuning brain circuits, whether to study them or to treat disease, is steadily becoming reality. With careful research and clinical translation, US-nano neuromodulation could in the future offer patients treatments that are high-impact but low-risk: for example, treating depression or epilepsy not with systemic drugs or invasive electrodes, but with a brief ultrasonic session guided by nanomedicine. Such capabilities would mark a significant leap forward in neuromodulation therapy, fulfilling the promise of precision medicine in neurology and psychiatry. The coming years will be crucial for turning these cutting-edge techniques into viable clinical tools, and the continued collaboration of engineers, biologists, clinicians, and regulatory agencies will be essential to safely unlock the full potential of ultrasound-assisted multimodal neuromodulation.

## Data Availability

Data sharing is not applicable to this article as no datasets were generated or analyzed during the current study.

## Abbreviations

BBB: Blood–Brain Barrier
DALYs: Disability-Adjusted Life-Years
DBS: Deep Brain Stimulation
tDCS: Transcranial Direct Current Stimulation
TMS: Transcranial Magnetic Stimulation
US: Ultrasound
I_SPTA_: Spatial Peak Temporal Average Intensity
I_SPPA_: Spatial Peak Pulse Average Intensity
PRF: Pulse Repetition Frequency
DC: Duty Cycle
MRI: Magnetic Resonance Imaging
FDA: Food and Drug Administration
TRP: Transient Receptor Potential
TWIK: Tandem of Pore Domains in a Weak Inward-Rectifying K+ Channel
TREK: TWIK-Related K+ Channel
FUS: Focused Ultrasound
GVs: Gas Vesicles
ML: Mechanoluminescent
ChR2: Channelrhodopsin-2
VTA: Ventral Tegmental Area
OFUS: Optically-generated Focused Ultrasound
SOAP: Soot-based Optoacoustic Pad
PDMS: Polydimethylsiloxane
NIR: Near-Infrared
EMG: Electromyography
PZT: Lead Zirconate Titanate
PVDF: Poly(vinylidene fluoride)
P(VDF-TrFE): Poly(vinylidene fluoride–trifluoroethylene)
BTNPs: Barium Titanate Nanoparticles
BNNTs: Boron Nitride Nanotubes
pMUT: Piezoelectric Micromachined Ultrasonic Transducer
PD: Parkinson’s Disease
TH: Tyrosine Hydroxylase
MAP2: Microtubule-associated Protein 2
GFAP: Glial Fibrillary Acidic Protein
SYN1: Synapsin I
PSD95: Postsynaptic density protein 95
CaMKII: Calcium/calmodulin-dependent protein kinase II
CaM: Calmodulin
PFOB: Perfluorooctyl Bromide
LGN: Lateral Geniculate Nucleus
PLGA: Poly(lactic-co-glycolic acid)
ZnS: Zinc Sulfide
ZnS:Ag,Co: Silver and Cobalt doped Zinc Sulfide
YFP: Yellow Fluorescent Protein
M2: Secondary Motor Cortex
DPPC: Dipalmitoylphosphatidylcholine
DPPA: Dipalmitoylphosphatidic Acid
MPEG5000 DPPE: Methoxy Polyethylene Glycol 5000 conjugated Dipalmitoylphosphatidylethanolamine
DFB: Decafluorobutane
DRN: Dorsal Raphe Nucleus
5-HT: 5-hydroxytryptamine
MoS_2_ NS: Molybdenum Disulfide Nanosheets
STN: Subthalamic Nucleus
SD: Sprague-Dawley (rats)
pDA: Polydopamine
NaV: Voltage-Gated Sodium Channel
KIR: Inward Rectifier Potassium Channel
